# scNucMap: mapping the nucleosome landscapes at single-cell resolution

**DOI:** 10.1093/bioinformatics/btaf324

**Published:** 2025-05-27

**Authors:** Qianming Xiang, Binbin Lai

**Affiliations:** Institute of Medical Technology, Peking University Health Science Center, Beijing 100191, China; Biomedical Engineering Department, Institute of Advanced Clinical Medicine, Peking University, Beijing 100191, China; Institute of Medical Technology, Peking University Health Science Center, Beijing 100191, China; Biomedical Engineering Department, Institute of Advanced Clinical Medicine, Peking University, Beijing 100191, China; Department of Dermatology and Venerology, Peking University First Hospital, Beijing 100191, China; State Key Laboratory of Molecular Oncology, Peking University International Cancer Institute, Beijing 1000191, China

## Abstract

**Motivation:**

Nucleosome depletion around cis-regulatory elements (CREs) is associated with CRE activity and implies the underlying gene regulatory network. Single-cell micrococcal nuclease sequencing (scMNase-seq) enables the simultaneous measurement of nucleosome positioning and chromatin accessibility at single-cell resolution, thereby capturing cellular heterogeneity in epigenetic regulation. However, there is currently no computational tool specifically designed to decode scMNase-seq data, impeding the generation of more precise and context-dependent insights into chromatin dynamics and gene regulation.

**Results:**

Here, we present scNucMap, a tool designed to leverage the unique characteristics of scMNase-seq data to map the landscapes of candidate nucleosome-free regions (NFRs). scNucMap demonstrated superior performance and robustness in cell clustering on scMNase-seq data compared to Signac and chromVAR across diverse sample compositions and data complexities, achieving higher overall accuracy and Kappa coefficients. Additionally, scNucMap identified significant TFs associated with nucleosome depletion at CREs at both single-cell and cell-cluster levels, thereby facilitating cell-type annotation and regulatory network inference. When applied to scATAC-seq data, scNucMap enriched standard analyses with complementary insights into nucleosome architecture, underscoring its cross‑modality versatility. Overall, scNucMap exhibits both high reliability and adaptability, making it an effective tool for analyzing scMNase-seq data and supporting multimodal studies, thereby illuminating the intricate relationship between regulatory networks and nucleosome positioning at single-cell resolution.

**Availability and implementation:**

scNucMap is available at https://github.com/qianming-bioinfo/scNucMap

## 1 Introduction

Nucleosomes are the fundamental units of chromatin in eukaryotes, each composed of a histone octamer and the DNA wrapped around it. Adjacent nucleosomes are connected by linker DNA of varying lengths. From an epigenetics perspective, DNA in nucleosome-free regions (NFR) is generally regarded as ‘open’ due to the absence of histones, which ensures accessibility to a series of regulatory factors, such as transcription factors (TFs) (Klemm *et al.* 2019). Consequently, nucleosome occupancy and positioning undergo dynamic alterations in response to the functional demands of cellular processes, reflecting the intricate regulation of chromatin structure in distinct physiological contexts (Brahma and Henikoff 2020).

Nucleosome occupancy and positioning affect gene expression within individual cells (Carter and Zhao 2021), serving as a regulatory mechanism manifested through rapid dynamic changes in nucleosome spacing. These alterations, driven by nucleosome disassembly or sliding along DNA, facilitate the spatial accommodation of protein binding. A series of techniques have been developed to directly map nucleosome positioning. Chromatin immunoprecipitation followed by deep sequencing (ChIP-seq) selectively enriches DNA fragments bound to these histone complexes (Park 2009, Fang *et al.* 2022). Methidiumpropyl-EDTA sequencing (MPE-seq) utilizes MPE-Fe(II) to detect core histones and infer nucleosome presence (Ishii *et al.* 2015). Among these methods, micrococcal nuclease sequencing (MNase-seq) remains the most widely utilized approach for analyzing nucleosome positions, as it provides unparalleled accuracy and resolution through the selective digestion of exposed linker DNA (Schones *et al.* 2008, Xu *et al.* 2022, Wernig-Zorc *et al.* 2024).

In response to the demand for detailed investigation at the cellular level, single-cell MNase-seq (scMNase-seq) was developed, empowering more precise mapping of chromatin accessibility in specific target contexts (Lai *et al.* 2018, Ren *et al.* 2022). Although scMNase-seq has demonstrated remarkable capability in distinguishing nucleosome positioning and chromatin accessibility under various conditions, a dedicated tool for analyzing and interpreting the resulting sequencing data remains unavailable. Existing tools designed for MNase-seq data, such as iNPS (Chen *et al.* 2014), DANPOS (Chen *et al.* 2013) and nucleR (Flores and Orozco 2011), operate at the level of bulk-cell analysis and fail to capture single-cell characteristics. Consequently, scMNase-seq data are typically analyzed using alternative tools, such as chromVAR (Schep *et al.* 2017) and Signac (Stuart *et al.* 2021), which are not tailored for this technique (Preissl *et al.* 2023). Such limited compatibility likely leads to suboptimal outcomes.

The nucleosome signal profile derived from scMNase-seq data resembles a mountainous landscape, with MNase-digested DNA fragments mapped around the nucleosome dyads forming peaks, and NFRs manifesting as valleys situated between them. The characteristics of these valleys—such as their depth, width, and steepness—can vary in response to the demands of gene expression. Valleys with a higher potential for protein binding tend to be deeper, broader, and flatter, in contrast to those with lower binding potential, where nucleosome fragments are more sparsely distributed. The variation in these valley landscapes provides insight into the relationship between the regulatory network and chromatin accessibility across different cell types.

Here, we present scNucMap, a tool specifically designed for mapping the landscapes of NFRs between adjacent nucleosomes. Leveraging scMNase-seq data, scNucMap enables the quantification and assessment of accessibility surrounding candidate transcription factor binding site (TFBS) centers. Each cell is characterized by the valley landscape measurements of a series of TFs, revealing differences in gene expression profiles, functional properties, and differentiation status. This facilitates comprehensive analyses, such as cell clustering and annotation at single-cell resolution. Overall, scNucMap not only distinguishes sample attributes for cell type identification through an epigenetic lens, but also uncovers potential cell-specific TFs that may contribute to future studies on regulatory networks, cellular mechanisms, and other relevant fields.

## 2 Materials and methods

### 2.1 Overall design of scNucMap

**Figure 1.**
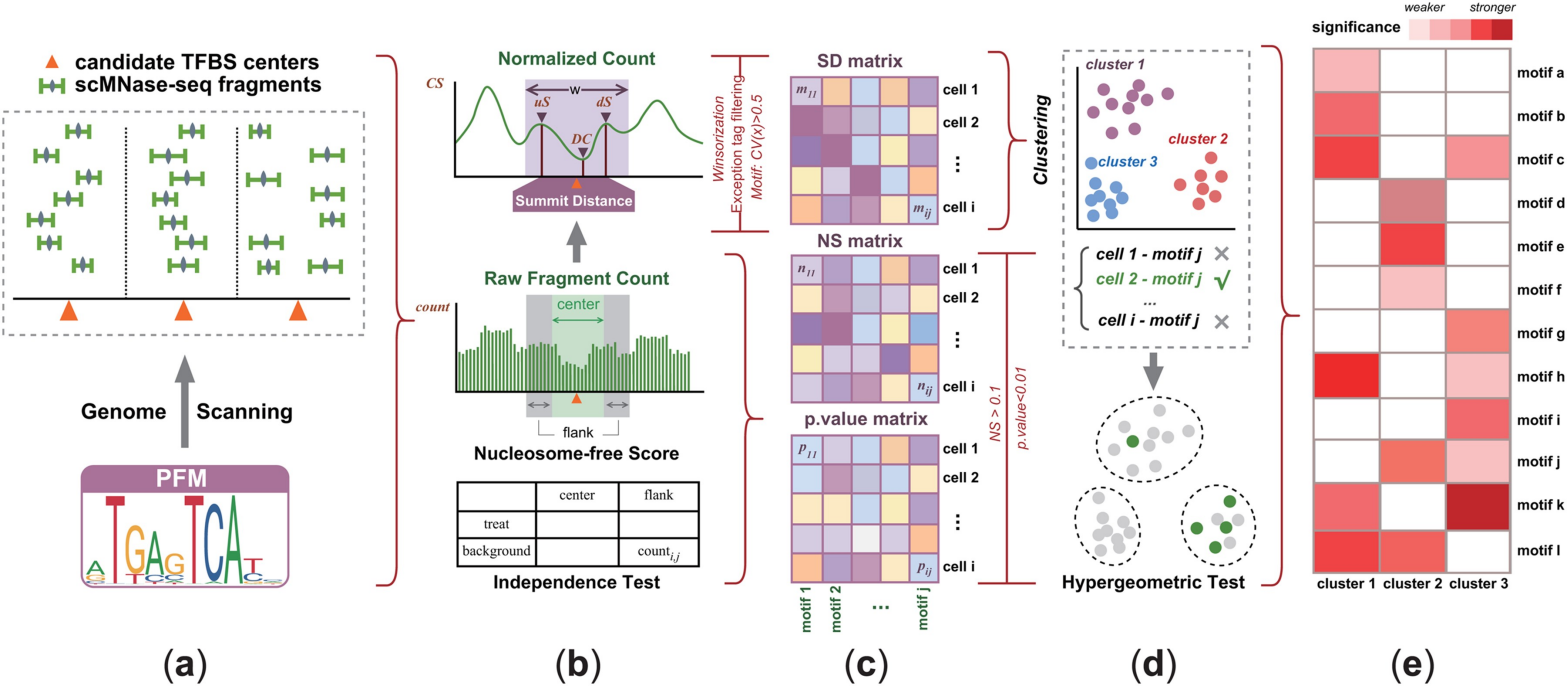
Overview of scNucMap. scNucMap maps the aggregated nucleosome landscape surrounding TFBS centers, thereby grouping single-cell samples into clusters and predicting active TF motifs at the single-cell or cell-cluster level. (a) Prediction of candidate TFBSs and counting of nucleosomal fragments. (b and c) Characterization of nucleosome landscapes using summit distances and joint tests integrating nucleosome-free scores and independence tests. (d and e) Identification of remarkable motifs.

scNucMap is designed for the analysis of scMNase-seq data, with two primary objectives: first, to cluster single cells into distinct groups from heterogeneous cell populations; and second, to identify significant TFs that orchestrate chromatin accessibility and nucleosome positioning at both the single-cell and cell group levels.scNucMap accepts two main inputs: nucleosomal fragments derived from scMNase-seq data for individual cells, and a set of candidate TFBS centers (Fig. 1a).

The method evaluates the aggregated nucleosome landscape surrounding TFBS centers by analyzing the distribution of nucleosome fragments. It calculates the summit distance (*SD*) between the nearest upstream and downstream nucleosomes relative to the TFBS center, enabling the assignment of single-cell samples to distinct clusters. For each single cell, the significance of TF binding possibility is assessed using a joint test that incorporates the nucleosome-free score (*NS*) and independence test (Fig. 1b and c). By linking cluster labels with motif activity predictions for each cell, scNucMap identifies cluster-specific motifs, thereby facilitating cell type annotation and regulatory network analysis (Fig. 1d and e).

### 2.2 Counting nucleosome fragments around obtained TFBS centers in open regions

TFBS could be derived from experimental data, such as ChIP-seq, or predicted computationally based on TF motifs. By default, scNucMap utilizes MOODS (Korhonen *et al.* 2017), a suite of algorithms for motif occurrence detection, to process the input position frequency matrices (PFMs) for TFBS identification. MOODS can scan the entire genome sequence for each PFM and output the positions of matching hits. As this prediction is based solely on sequence similarities, hits located outside established accessible regions, such as DHS or ATAC-seq peak regions, were filtered out to enhance the reliability of the predictions.

The midpoint of each scMNase-seq fragment, ranging from 140 to 180 bp, was considered the nucleosome position and counted once per bin (with the default bin size set to 1 bp). For a specific TF motif towards a single cell, the raw count vectors of each candidate TFBS were aligned at the center, and then summed to generate a “piled-up” vector. This “piled-up” vector represents the piled-up nucleosome position landscapes across pooled regions centered on the TF motif. The length of this vector was determined by dividing the window size (with a default range of 400 bp upstream and downstream) by the bin size. Both the window and bin sizes were set based on considerations of typical nucleosome spacing and the spatial constraints required for TF binding. These piled-up vectors, which reflect the aggregated distribution of nucleosome fragments, were subsequently used in the calculation of metrics.

### 2.3 Summit distance measures region accessibility

To analyze nucleosome landscape around TFBS centers, for a given TF motif, we called the nucleosome positions relative to each TF motif hit within open chromatin region, generate a composite nucleosome positioning profile by summing across all instances of the TF motif hit, and then calculate positioning scores to quantify nucleosome density patterns.

Specifically, to quantify the consistency of nucleosome fragments and normalize the counts at each site, positioning scores (Gaffney *et al.* 2012) were calculated for each piled-up vector:

(1) $$S_{i}=\frac{\sum_{j=i-15}^{i+15}x_{j}}{\sum_{j=i-100}^{i+100}x_{j}}$$

The positioning score *S_i_* represents the proportion of nucleosome fragment midpoints located within a 31 bp region and a surrounding 201 bp region centered on site *i*. *x_j_* is the number of midpoints at site *j*. We then defined center-weighted score (*CS*) as the normalized nucleosome count at site *i*, where *w_j_* is a symmetrically distributed distance-spreading weight (Lai *et al.* 2018):

(2) {CS(i)=∑j=-7373Si+jwjwj=e-(j/20)2/2

Summit distance (*SD*) is the key measurement for assessing the relative accessibility around TFBS centers. The local maxima of the *CS* vector, both upstream and downstream relative to the TFBS center (default range of 200 bp on each side, accounting for the nucleosome DNA length plus linker DNA length), were regarded as the assumed dyad positions, referred to as “summits’.” Their indices are denoted as *uS* and *dS* in Equation (1). Consequently, this count-weighted distance is defined as:

(3) {SD=∑i=uSdSdi×ni∑i=uSdSnidi=|idxi-idxDC|idxDC=idxmin⁡{[-r,+r]}

The dynamic center (*DC*) was defined as the bin index of the local minimum value in the *CS* vector within a flanking region from *−r* to +*r* around the center (Fig. 1b). The default value of *r* was set to 20 to capture the potential shifts between actual binding sites and the motif center. The inter-bin distance was defined as the absolute difference between the indices of bins, where *idx_i_* represents the index of bin *i*. Let *n_i_* denote the fragment count of bin *i*, the weighted distance was independently calculated for the upstream and downstream regions relative to the *DC* and subsequently summed to obtain the *SD*. Utilizing the piled-up vectors from each cell as input, a cell-motif matrix *M* was constructed, where *m_i, j_* represents the *SD* value of cell *i* for motif *j*. A higher *SD* value indicated an increased concentration of nucleosome fragments near the summits, suggesting greater accessibility of the valley region for protein binding on exposed DNA.

To accurately characterize the landscape surrounding TFBS centers, the binary “fragment hits” status of each bin was recorded as a quality control metric to assess sequencing depth and coverage, where 0 indicates the absence of fragments and 1 indicates their presence. Piled-up vectors with a proportion of hit bins below the threshold of 5% (default) were assigned an *SD* value of 0 as an exception tag. Subsequently, scNucMap filtered cell samples and motifs based on the proportion of exception tags within rows and columns, applying default cutoffs of 70% and 90%, respectively.

### 2.4 Cell clustering

The cell-motif matrix *M* encapsulated variations in potential TF binding activities across individual cells, serving as the foundation for cell clustering. To minimize the impact of outliers, the elements in *M* were winsorized within each motif column, with extreme values replaced by those at predefined percentile thresholds.

Motifs exhibiting high variation were identified by calculating their coefficient of variation (*CV*), where *x_i_* stands for the SD value towards cell *i* for a given motif, $\bar{x}$ denotes the mean SD value within the motif column, and *n* is the total number of cells:

(4) $$CV(x)=\frac{\sqrt{\frac{1}{n-1}\sum_{i=1}^{n}\left(x_{i}-\bar{x}\right)}}{\bar{x}}$$

Only motifs with a *CV* exceeding the threshold (default set at 0.5) were retained for clustering, demonstrating that their SD values exhibit pronounced fluctuations across individual cells. Both tree-based and graph-based methods are available to illustrate the heterogeneity and similarity among cells based on the quality-filtered matrix *M′*.

### 2.5 Remarkable TF motif identification

TFs with high predicted binding activity demonstrate similar characteristic profiles: their NFR valleys are typically broader and flatter, flanked by steeper nucleosome peaks. These motifs could be identified at both the single-cell and cell-cluster levels, each serving distinct purposes in decoding cellular functions at varying levels of resolution, with partially shared approaches.

Building on the common patterns observed in nucleosome fragment distributions, a joint filtering process was applied using the nucleosome-free score (*NS*) (Lai *et al.* 2018), combined with an independence test. This approach evaluates the accessibility of a specific TF in relation to the genomic background. The nucleosome-free score (*NS*) is defined as:

(5) {NS=log2(FbFt)F=ncenternflank+ncenter

*n_center_* denotes the sum of fragment counts within ±100 base pairs of the region center, whereas *n_flank_* corresponds to the aggregated fragment counts within the 100–200 base pair range from the center, on both the upstream and downstream sides (Fig. 1b). *F_b_* and *F_t_* stand for the fraction of background and treatment regions, respectively. *NS* is calculated as the log2 ratio value of these two fractions. While *NS* was initially designed for single-cell data, it remains applicable to pooled samples, particularly in cases of suboptimal single-cell coverage or when such data are unavailable.

While *NS* evaluates the relative enrichment of nucleosome fragments between treatment and background regions surrounding the centers, the significance of this distribution preference remains unclear. To address this, we constructed a 2 × 2 contingency table, with rows representing center and flank regions and columns indicating treatment and background sources. Chi-square tests or Fisher’s exact tests were performed as appropriate. The null hypothesis assumed no difference in the distribution between the center and flank regions attributable to the source.

We applied the default cutoff thresholds of *NS* > 0.1 and an independence test *P*-value < 0.01 to filter TFs with significant binding activity at single-cell level. A hypergeometric test was then conducted to identify cluster-specific active TFs, based on the cumulative frequency of each motif within a cluster, thus capturing the cell-motif relationships (Fig. 1d and e). Downstream analyses, such as Gene Ontology (GO) enrichment, were subsequently performed to validate these findings with biological evidence.

### 2.6 Generalization to scATAC-Seq data

We extended the application of scNucMap to scATAC-seq data, with reference to Signac’s motif-analysis workflow (Stuart *et al.* 2021). For each cell type, peaks were called on pooled fragments and merged into a unified, non-overlapping peak set to define accessible regions for downstream analysis. Signac-defined mononucleosomal fragments (147–294 bp) were then extracted and provided as input to scNucMap.

Since Signac wraps chromVAR to compute bias-corrected motif accessibility z-scores using the RunChromVAR function, and given the near-perfect clustering performance of chromVAR described in Section 3.4, comparisons of identified TF sets were restricted to scNucMap and chromVAR. We followed the workflow employed in Signac: based on these *z*-scores, a Wilcoxon rank-sum test was performed for each motif to compare its *z*-score distribution in the target cluster against that in all other clusters. Motifs exhibiting consistently higher *z*-scores in the target cluster were considered significantly active.

### 2.7 Data overview

We downloaded three scMNase-seq datasets (Datasets 1–3) for benchmarking and one scATAC-seq dataset (Dataset 4) to evaluate the generalizability of scNucMap (Figs S1–S6, available as supplementary data at *Bioinformatics* online and Tables S1–S7, available as supplementary data at *Bioinformatics* online).

- Dataset 1: 48 NIH3T3 single cells, 203 mouse embryonic stem cells (ESCs) and 288 mouse naive CD4 T cells under GEO accession number GSE96688, 539 cell samples in total (Lai *et al.* 2018).
- Dataset 2: 331 wild type early innate lymphoid progenitor (EILP-WT), 250 Tcf7-KO EILP (EILP-KO) and 251 wild type innate lymphoid cell precursor (ILCP-WT) under GEO accession number GSE142468, 832 cell samples in total (Ren *et al.* 2022).
- Dataset 3: Mouse preimplantation embryos, comprising 1-cell stage (*n* = 5), 2-cell stage (*n* = 20), 4-cell stage (*n* = 13), 8-cell stage (*n* = 22) and morula stage (*n* = 31), 91 cell samples in total. Data were downloaded from Genome Sequence Archive (https://ngdc.cncb.ac.cn/gsa/) under accession number CRA014259.
- Dataset 4: 384 CD4^+^ T cells (CD4), 384 cardiac progenitor cells (mCPC), 384 embryonic stem cells (mESC), and 384 skin fibroblasts (mSF) from mouse single-cell ATAC-seq data with ENA accession number ERP108537. CD4 and mCPC were randomly downsampled to 384 cells to match the cell number of mSF and mESC, yielding 1536 cells in total (Chen *et al.* 2018).

In addition, a total of 835 non-redundant PFMs of mouse and human for motif matching were downloaded from the JASPAR database (Rauluseviciute *et al.* 2024). DHSs obtained from ENCODE (https://www.encodeproject.org/) were utilized for TFBS filtering and served as the chromatin accessibility background across the three scMNase-seq datasets. For each dataset, only the TFBSs that intersected with the union accessible regions, each extended by 200 bp from the center, were retained for subsequent analysis.

### 2.8 Clustering performance evaluation and comparison

We assessed the clustering results using overall accuracy and Kappa coefficient derived from the confusion matrix. Overall accuracy reflects the proportion of correctly assigned mappings, providing a straightforward measure of model performance. The Kappa coefficient evaluates the agreement between clustering results and true labels, accounting for chance agreement and adjusting for sample size imbalance within clusters, with values ranging from −1 to +1. A Kappa value of 0 indicates no better agreement than random assignment, whereas higher values signify stronger agreement.

For comparative analysis, we selected two widely used tools, Signac and chromVAR. Clustering was conducted using each tool’s default procedures and parameters. All shared input data were kept consistent, including scMNase-seq fragment data, open chromatin region data, and PFMs. To visualize the population structure of single-cell samples in two dimensions, we employed uniform manifold approximation and projection (UMAP) as the dimensionality reduction method, capturing the complexity of cell-motif relationships.

Subsequently, we conducted robustness tests involving multiple iterations of random sampling with predefined proportions for each class. In each iteration, a confusion matrix was generated with consistent dimensions to allow for element-wise summation, thereby producing an overall confusion matrix for a given sampling proportion.

Signac utilizes the Louvain and Leiden algorithms, which do not predetermine the number of clusters. Instead, the clustering outcome depends on adjusting the resolution parameter according to the sample data. In comparison, chromVAR uses hierarchical clustering to construct a dendrogram, capturing the nested relationships between samples based on pairwise distances. Distinct clusters can then be extracted, making hierarchical clustering a practical and flexible method for generating fixed-dimensional confusion matrices.

**Figure 2.**
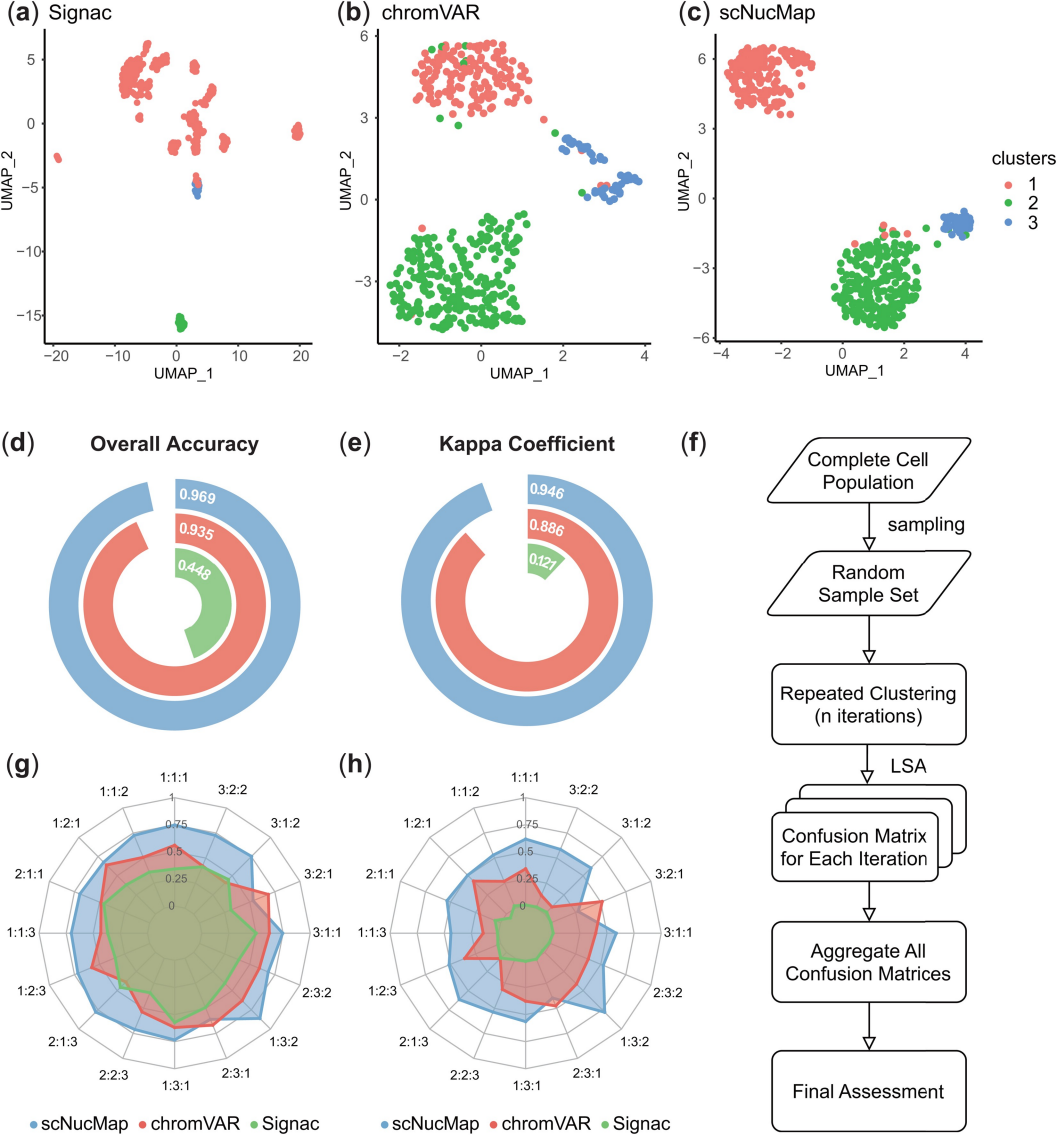
Comparison of clustering performance among Signac, chromVAR, and scNucMap. (a–c) UMAP projections of single-cell samples from Dataset 1, processed using the three tools, with cluster labels indicated. (d and e) Performance assessment, corresponding to the results shown in (a–c), based on overall accuracy and the Kappa coefficient. (f) Overview of the robustness test procedure. (g and h) Radar plots assessing the performance of the three tools across clustering metrics at various sampling ratios. Each dimension represents the sampling ratio of ESC: CD4: NIH3T3.

The procedures for robustness testing are shown in Fig. 2f. With a specific proportion, all cells from Dataset 1 were randomly sampled 100 times and used as the same input for the three tools. Each tool was executed according to its internal pipeline, proceeding to the clustering step. At this point, the matrices used for clustering were extracted and subjected to hierarchical clustering with a target of three clusters.

In each iteration, the Linear Sum Assignment (LSA) algorithm, utilizing the Hungarian method, was applied to explore potential mappings between clustering labels and ground truth labels. The mapping yielding the highest overall accuracy was selected to construct the optimized confusion matrix. All matrices generated across iterations were aggregated into an overall confusion matrix, which served as the basis for calculating overall accuracy and the Kappa coefficient.

## 3 Results

### 3.1 Superiority of scNucMap in clustering performance based on scMNase-seq data over existing tools

The efficacy of single-cell omics methods largely relies on the capability of dissecting discrete cell types by clustering and annotation in silico from a mixed population. To evaluate the performance of scNucMap in clustering different cell types from scMNase-seq data with a mixed population, we applied scNucMap to simulated benchmarking datasets, which were generated by mixing scMNase-seq datasets from NIH3T3 single cells, ESCs, and naive CD4 T cells from Dataset 1, encompassing 539 single cells (Lai *et al.* 2018). After all the quality control and feature filtering procedures, approximately 91.11% of cells and 37.72% of motifs from Dataset 1 were retained for subsequent analysis.

Given the absence of specialized tools tailored to scMNase-seq data characteristics, we compared scNucMap against two widely adopted tools, Signac and chromVAR, both of which infer chromatin accessibility from single-cell data. Clustering was performed using the default parameters of Signac and chromVAR on the entire set of 539 cells from Dataset 1, ensuring consistency with the data processed by scNucMap. The open region data, which consisted of the extended union DHS set (200 bp), and the PFMs were uniformly applied across all tools.

After quality control, Signac, chromVAR, and scNucMap retained 514, 477, and 479 cells, respectively for clustering. Notably, scNucMap showed the best performance compared to the other two tools. The UMAP visualization of scNucMap’s clustering results exhibited compact and well-defined intra-cluster groupings with clear and distinct inter-cluster boundaries (Fig. 2a–c). Quantitatively, scNucMap achieved an overall accuracy of 0.969, showing an improvement over chromVAR (0.935), and both markedly surpassing Signac (0.448) (Fig. 2d). Similarly, scNucMap achieved a Kappa coefficient of 0.946, outperforming chromVAR (0.886) and significantly exceeding Signac (0.121) (Fig. 2e).

To further demonstrate scNucMap’s advantage across varying data volumes and levels of class imbalance, robustness tests were conducted comparing the three tools. A total of 16 sampling proportions were evaluated, ensuring equal consideration of potential class biases. Overall, scNucMap consistently outperformed the other tools in both overall accuracy and Kappa coefficient (Fig. 2g and h; Figs S7–S9, available as supplementary data at *Bioinformatics* online). Specifically, scNucMap demonstrated a clear advantage over Signac across all proportion scenarios. In only two instances, scNucMap showed decreases of 0.058 and 0.152 in overall accuracy, and 0.079 and 0.240 in the Kappa coefficient compared to chromVAR. However, these decreases were smaller than the median performance advantages scNucMap maintained over chromVAR, which were 0.199 and 0.268 in overall accuracy and Kappa coefficient, correspondingly.

Collectively, these findings underscore scNucMap’s superior capability to produce accurate and reliable clustering outcomes. Additionally, scNucMap demonstrates robust performance across varying sample compositions and maintains scalability amidst diverse complexities (Fig. S10, available as supplementary data at *Bioinformatics* online, Tables S8 and S9, available as supplementary data at *Bioinformatics* online), highlighting its edge over existing tools in the clustering analysis of scMNase-seq data.

### 3.2 scNucMap identified remarkable TFs exhibiting cluster-specific features

TFs with potentially high binding activities were identified by scNucMap across individual cells in Dataset 1 using the default threshold. Integrating clustering analyses, we highlighted TFs exhibiting significant cluster-specific characteristics, corroborated by compelling biological evidence.

**Figure 3.**
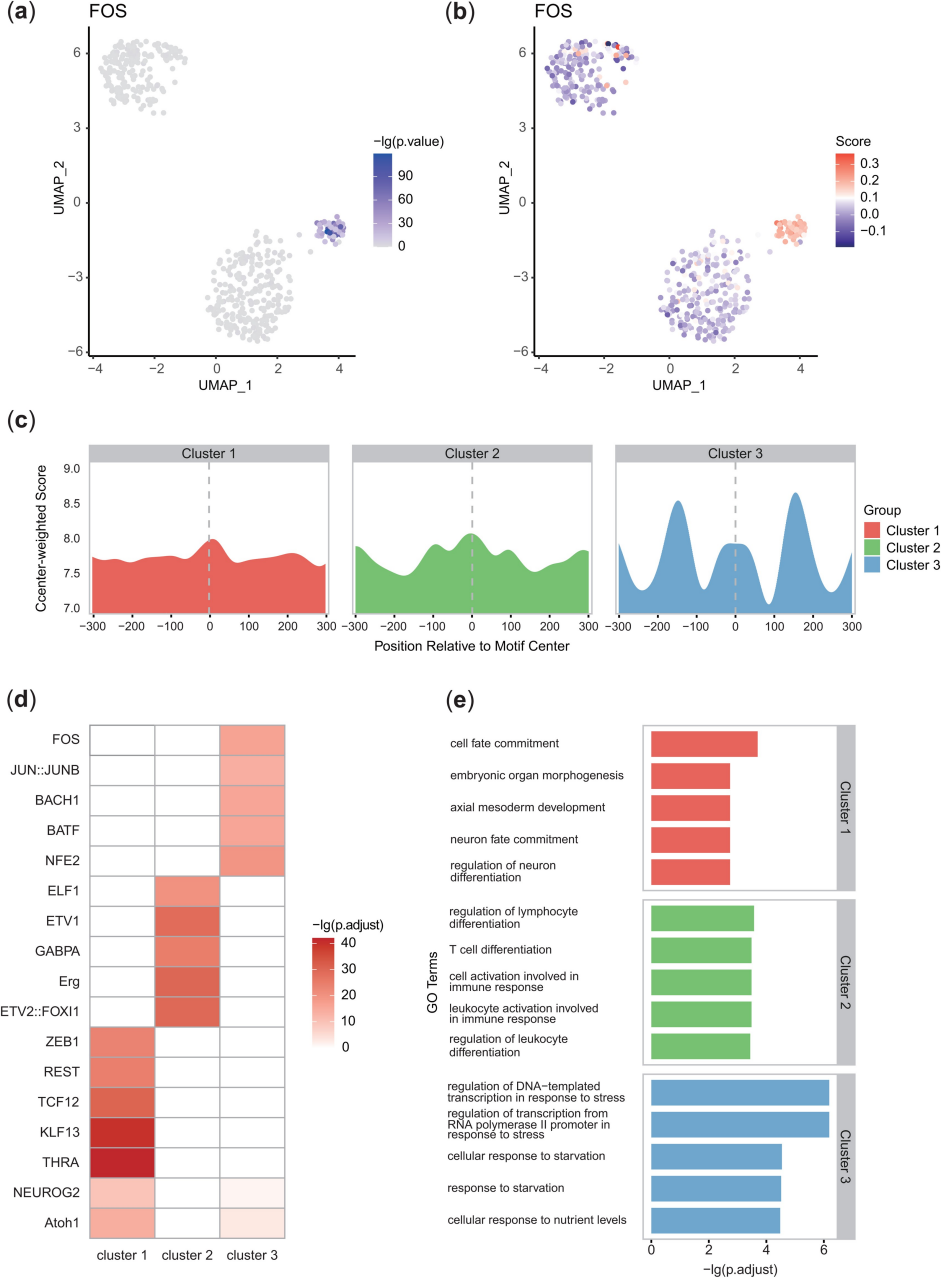
Remarkable TF motifs identified by scNucMap exhibit distinct patterns consistent with biological expectations. (a and b) Results of the independent test and nucleosome-free score for FOS, projected onto sample points in UMAP space. (c) Average profiles of centered TFBSs of FOS. (d) Cluster-specific TF motifs identified by scNucMap with high confidence. (e) Top GO enrichment analysis results for biological process terms based on cluster-specific TFs.

FOS emerged as a TF with prominent cluster-specific binding activities. The hypergeometric test revealed that FOS binding was significantly elevated in cells from cluster 3 compared to those in clusters 1 and 2 (adjusted *P*-value = 3.68×$10^{-17}$). We subsequently mapped the *P*-values from the independence test and *NS* onto individual samples within the UMAP space (Fig. 3a and b), uncovering distinct pattern distributions across the clusters. As shown in Fig. 3c, these differences stem from variations in the landscape shaped by nucleosome fragment distributions.

Upon comparing ground truth labels with cluster assignments, we found that cluster 3 was exclusively composed of NIH3T3 cells. As the subunit of AP-1 transcription factor complex together with JUN family, the expression and regulation of the FOS family are associated with the cell cycle progression in NIH3T3 cells (Lallemand *et al.* 1997), and play a pivotal role in cellular fibrosis (Schulien *et al.* 2019, Qi *et al.* 2024).

Besides FOS, other TFs that emphasize distinct characteristics unique to each cluster were also detected by scNucMap (Fig. 3d; Fig. S11, available as supplementary data at *Bioinformatics* online). To comprehensively assess these remarkable TFs, we performed GO enrichment analysis on each set of cluster-specific TFs. According to the promising clustering accuracy, cluster 1 and 2 are associated with ESCs and naive CD4 T cells, respectively. The biological process enrichment results aligned closely with the corresponding cell types (Fig. 3e). Enriched GO terms of cluster 1 suggest a strong association with cell fate commitment and embryonic organ morphogenesis, whereas the significant terms for cluster 2 were primarily linked to T cell differentiation. For cluster 3, the enriched terms aligned with the biological characteristics of NIH3T3 cells, which are known for their roles in transcriptional regulation and stress response, reflecting their capacity to dynamically regulate gene expression and adapt to cellular processes. These biological evidences further substantiate the reliability of scNucMap in identifying remarkable TFs.

### 3.3 scNucMap identified dynamic TFs activity in early ILC progenitor differentiation and mouse preimplantation embryo development

scNucMap identified key motifs demonstrating distinctive population patterns in samples from Dataset 1, supported by established research. To assess the broader applicability of our method, we extended its analysis to Dataset 2 and 3, at the pooled cell level, corresponding to their respective cell types.

**Figure 4.**
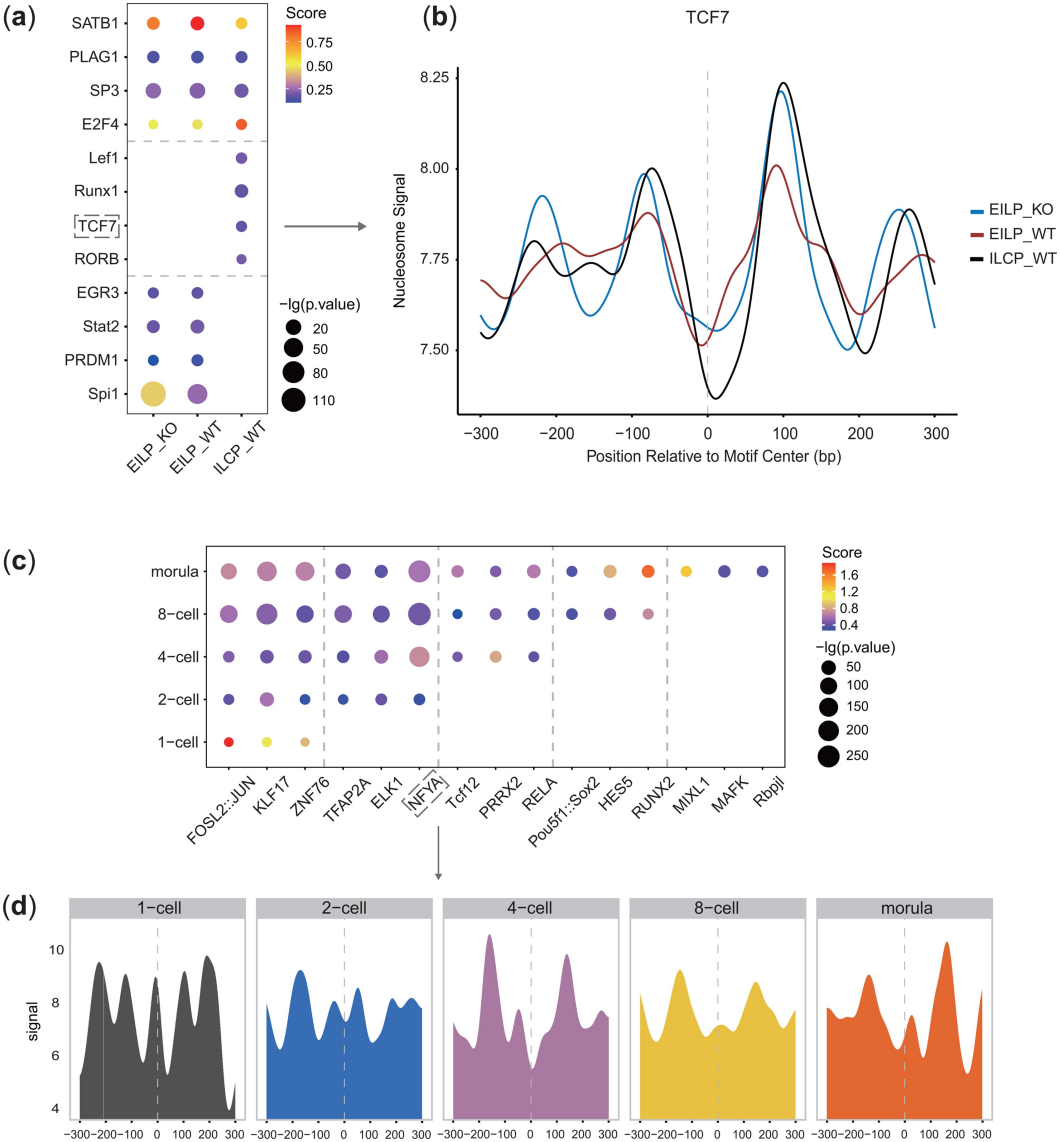
Average profiles reveal that the predicted TF activity trends are consistent with filtering metrics across datasets, with key TFs corroborated by prior research. (a) Selected TFs identified by scNucMap demonstrated either shared or cell-type-specific activities in EILP_KO, EILP_WT, and ILCP_WT from Dataset 2. The activity trends of Lef1, TCF7, and Spi1 match those reported in previous studies. Sites without data points indicate failure in joint filtering. (b) Average profiles of TCF7 across different cell types, presented as representative cases. (c) Selected TFs identified by scNucMap exhibited increased activities at specific developmental stages from 1-cell to morula from Dataset 3. The nucleosome occupancy trends of NFYA and Pou5f1::Sox2 (Oct4) align with findings from previous studies (Lu *et al.* 2016). (d) Average profiles of NFYA across various cell types, presented as representative examples.

Consistent with the published study (Ren *et al.* 2022), scNucMap detected high binding activity for TCF7 (encoding TCF-1) and Lef1 in wild-type ILC progenitor samples (ILCP-WT), whereas no significant activity was observed in wild-type early ILC progenitor samples (EILP-WT) and *Tcf7*-knocked-out early ILC progenitor samples (EILP-KO) (Fig. 4a and b). Furthermore, Spi1 (PU.1) was predicted to be active in both EILP-WT and EILP-KO, but exhibited reduced activity in ILCP-WT. These results highlight the distinct roles of these TFs in the epigenetic and transcriptional regulation of early ILC progenitor differentiation.

Additionally, a previous study (Lu *et al.* 2016) identified the roles of Nfya and Oct4 (encoded by *Pou5f1*) in the establishment of DHSs at the 2-cell and 8-cell stages, respectively. Notwithstanding the relatively limited coverage and sequencing depth of Dataset 3, our results corroborate their findings. Specifically, we observed that Nfya binding activity commenced at the 2-cell stage and persisted through the morula stage, whereas Pou5f1::Sox2 and RUNX2 exhibited significant activity from the 8-cell to the morula stage (Fig. 4c and d), and their increased activity from the 4-cell to 8-cell stage was independently validated by TOBIAS analysis (Bentsen *et al.* 2020) using a mouse embryo ATAC-seq dataset (GSE66581) (Fig. S12, available as supplementary data at *Bioinformatics* online).

The concordance between our results and published studies further underscores the versatility and dependability of scNucMap, reinforcing our confidence in its ability to identify candidate key TFs. While several TFs have been previously validated in the literature, many others—though not yet definitively confirmed as markers or consistently active across all cell types or stages—exhibited significant patterns in our analysis (Fig. 4a and c). For instance, Runx1 was predicted to be significantly more active in ILCP-WT compared to EILP-KO and EILP-WT, whereas the activity of PRDM1 diminished from EILPs to ILCPs. Established studies have demonstrated that Runx1 is essential for hematopoietic stem cell development and their differentiation into both myeloid and lymphoid lineages (Koh *et al.* 2013, Li *et al.* 2021), while PRDM1 is crucial for promoting B cell differentiation and T cell maturation, and serves as a key regulator in dendritic cell maturation and T cell priming (Nadeau and Martins 2021).

Thus, scNucMap provides targeted insights that can inform future research directions from an analytical perspective, shedding light on novel regulatory mechanisms and identifying potential key TFs involved in cellular differentiation and development.

### 3.4 scNucMap deciphered regulatory landscapes embedded within nucleosome-length fragments from scATAC-seq data

In addition to the primary use in analyzing MNase-seq data, scNucMap also leverages nucleosome-length fragments from ATAC-seq to depict the nucleosome landscapes surrounding TFBS centers, thereby offering complementary insights into regulatory networks associated with specific cell clusters. This contrasted with conventional ATAC-seq analysis, which mainly focuses on subnucleosomal fragment distributions.

We demonstrated this complementarity by comparing significantly active TFs identified at the cluster level using the same tools. As Signac employs chromVAR to compute motif activity scores via the RunChromVAR function, the comparison was conducted only between scNucMap and chromVAR.

**Figure 5.**
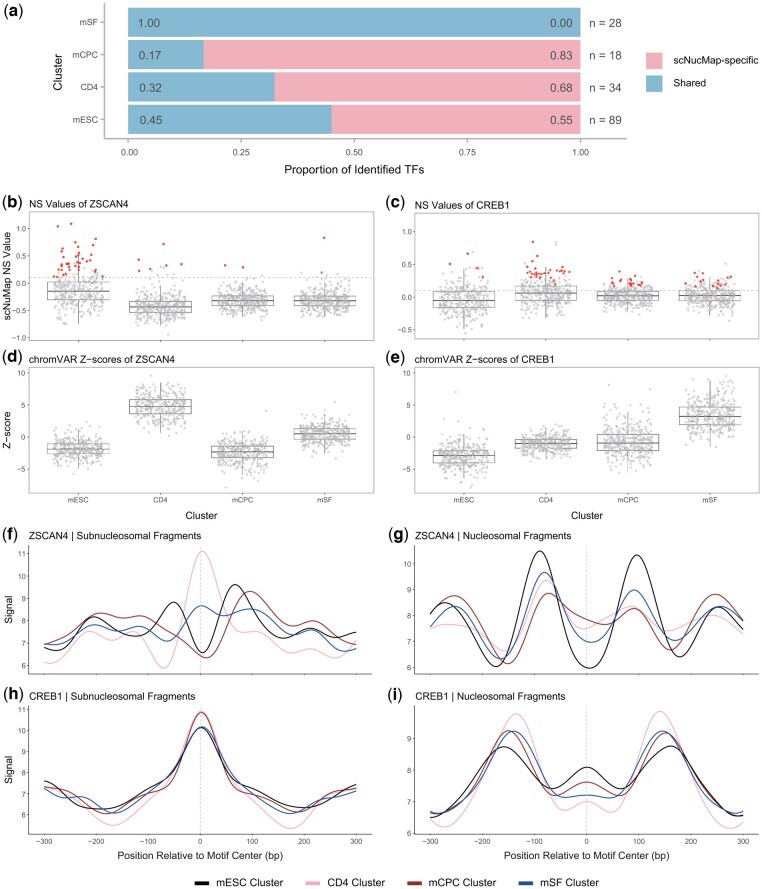
Active TFs identified by scNucMap are consistent with published studies and complement chromVAR‑derived results. (a) Stacked bar chart showing the proportions of TFs uniquely identified by scNucMap and those shared with chromVAR across the mESC, CD4, mCPC and mSF clusters. Proportion values are labeled within bars; total TF counts (n) are indicated to the right. (b and c) Boxplots of *NS* values for ZSCAN4 (b) and CREB1 (c) across clusters. Samples meeting the default joint‑test criteria (*NS* > 0.1 and independence test *P* < 0.01) are highlighted in red. The grey dashed line marks *NS* = 0.1. (d and e) Boxplots of chromVAR‑derived z‑scores for ZSCAN4 (d) and CREB1 (e) across clusters. (f–i) Average profiles of subnucleosomal (f and h) and nucleosomal (g and i) fragments across clusters for ZSCAN4 (f and g) and CREB1 (h and i).

We first performed cell clustering on Dataset 4. scNucMap achieved an overall accuracy of 86.59%, whereas chromVAR reached 99.37%, indicating near-perfect delineation of the cell populations. This was expected, as the clustering metric in scNucMap was not originally intended for ATAC-seq data. To improve downstream TF activity inference, scNucMap adopted the optimal clustering assignments from chromVAR. For clarity in linking TF activity to cell identity, clusters were subsequently annotated based on the predominant cell type in each group (e.g. the mESC cluster) (Fig. 5a; Tables S10–S13, available as supplementary data at *Bioinformatics* online).

In the mESC cluster, scNucMap uniquely identified TFs with critical roles in maintaining pluripotency and genome stability. FOXP1 supports self-renewal and the maintenance of stemness while stimulating OCT4 and NANOG (Gabut *et al.* 2011). ZFP57 regulates parent-specific gene expression through DNA methylation at imprinting sites (Anvar *et al.* 2016). ZSCAN4, a key modulator of telomere maintenance and genome integrity in 2-cell-like mESCs (Dan *et al.* 2017), was significantly overrepresented, as determined by hypergeometric enrichment analysis in scNucMap (Fig. 5b, d, f, and g).

Notably, scNucMap and chromVAR jointly contributed to a more comprehensive regulatory network that integrates extracellular signals and guides cell fate decisions in CD4^+^ T cells. TFs specifically identified by scNucMap were primarily from the CREB/ATF, AP-1, and IRF families, all of which are well-established regulators of T cell activation and differentiation (Tailor *et al.* 2006, Wen *et al.* 2010, Yamazaki *et al.* 2017). While other members of these families were also widely detected by chromVAR, the complementarity between the two tools became evident as scNucMap additionally captured key TFs that might otherwise be missed. For instance, CREB1, CREM and ATF-1 are key downstream effectors in the cAMP–PKA signaling pathway, which regulates several CD4^+^ T cell differentiation processes, including IL-2 and IL-4 production (Wang *et al.* 2017, Wang *et al.* 2024). CREM and ATF-1 were detected by both tools, whereas CREB1 was exclusively identified by scNucMap (Fig. 5c, e, h, and i).

Furthermore, among the 15 TFs specifically identified by scNucMap in the mCPC cluster, 13 belonged to the ETS family (Fig. 5a). ETS TFs play critical roles in hematopoietic and vascular generation (Liu *et al.* 2015), and interact with GATA4—a canonical marker TF involved in cardiac development—in regulating cardiac lineage commitment (Schachterle *et al.* 2012, Zhou *et al.* 2022). In the mSF cluster, all TFs identified by scNucMap were a subset of those detected by chromVAR. These TFs are mainly implicated in fibroblast activation (Luo *et al.* 2024), skin inflammation and fibrosis (Bergmann *et al.* 2025) and skin aging (Wang *et al.* 2021, Wang *et al.* 2022).

By capturing nucleosome occupancy patterns typically obscured in conventional ATAC-seq analyses, scNucMap complements standard approaches, underscoring the value of integrating chromatin accessibility and nucleosome positioning insights to unravel gene regulatory circuitry. By repurposing nucleosomal fragments that are typically masked from conventional ATAC-seq analysis workflows, scNucMap helped unveil latent chromatin features and yielded a richer, cell type-resolved portrait of TF engagement and chromatin organization.

## 4 Discussion

scMNase-seq has demonstrated exceptional capabilities in exploring and unraveling molecular mechanisms from an epigenetic perspective by analyzing nucleosome architecture within individual cells. This approach uncovers features that might remain obscured in bulk population analyses (Kong *et al.* 2022), facilitates precise cell type identification, and elucidates subtle regulatory mechanisms. Understanding heterogeneity at the single-cell level, as well as the relationship between chromatin landscapes and gene expression regulation, is crucial for deciphering complex biological processes such as development, differentiation, and disease progression (Baldi 2019).scNucMap evaluates the relative accessibility around TFBS centers by calculating summit distance for each cell to each motif based on scMNase-seq data, and has been further generalized to nucleosome-length fragments derived from ATAC-seq. A joint test integrating both nucleosome-free scores and independence tests is applied to identify TFs with significantly altered binding potential at both single-cell and pooled cell levels, thereby linking them to the expression of associated genes. TF characteristics—encompassing both shared and cluster‑specific activities—are aggregated across groups to construct group‑level regulatory networks, facilitating cell type annotation. While fundamentally designed for single-cell analysis, our method can adapt to bulk data when single-cell resolution is unavailable. However, single-cell analysis uniquely enables cell-type-specific TF prediction in heterogeneous samples and avoids confounding effects from population averaging. Bulk analysis remains suitable only for homogeneous populations where cellular resolution is unnecessary.

Due to the absence of tools tailored to the specific properties of scMNase-seq data, we compared scNucMap with two widely used scATAC-seq analysis tools, Signac and chromVAR, both compatible with scMNase-seq. However, the inherent differences between capturing accessible regions and nucleosome-occupied DNA necessitate customized analytical strategies. As our comparisons showed, scNucMap outperformed the other tools in both overall accuracy and Kappa coefficient, demonstrating superior predictive reliability and stronger agreement with true classifications beyond random chance. Furthermore, scNucMap’s robust performance across a range of sampling proportions ensures reproducibility under varying cell compositions and emphasizes the need to consider sequencing-specific properties for optimal results.

Analogous to how diverse geological forces converge to form real-world landforms, integrating multi-omics data with scMNase-seq promises a more precise and vivid portrayal of the chromatin landscape. Our preliminary extension to scATAC‑seq data demonstrated that scNucMap’s results complement those from chromVAR and align with established studies, underscoring its value in refining chromatin landmark identification. Furthermore, leveraging recent advances could lead to improved accuracy of scNucMap. Although we minimized the influence of pioneer TF by intersecting the predicted TFBS with chromatin accessibility regions, their ability to bind both open and nucleosome‑occupied DNA (Carminati *et al.* 2024, Huyghe *et al.* 2024) could not be fully excluded. Future work will construct integrative models that exploit multimodal data and account for pioneer TFs’ unique properties to build a comprehensive framework for elucidating chromatin architecture (Grand *et al.* 2024, Almassalha *et al.* 2025). Such models are poised to reveal latent insights into the interplay between epigenetic regulation and gene expression.

## Supplementary Material

btaf324_Supplementary_Data

## Data Availability

scNucMap is available at https://github.com/qianming-bioinfo/scNucMap
