# Supplementary material for: scNucMap: mapping the nucleosome landscapes at single-cell resolution
Source: Bioinformatics. 2025 May 27;41(6):btaf324. doi: 10.1093/bioinformatics/btaf324 (PMC12202765; doi:10.1093/bioinformatics/btaf324)
Supplement: btaf324_Supplementary_Data [file btaf324_supplementary_data.zip › scNucMap_Supplementary_Materials.pdf]

Supplementary Materials for "scNucMap: mapping the nucleosome landscapes at single-cell resolution"

Qianming Xiang[1,2] and Binbin Lai[1,2,3,4*]

[1]Institute of Medical Technology, Peking University Health Science Center, Beijing, 100191, China
[2]Biomedical Engineering Department, Institute of Advanced Clinical Medicine, Peking University, Beijing, 100191, China
[3]Department of Dermatology and Venerology, Peking University First Hospital, Beijing, 100191, China
[4]State Key Laboratory of Molecular Oncology, Peking University International Cancer Institute, Beijing, 1000191, China
[*]Corresponding author: laib@bjmu.edu.cn

1 Summary of nucleosomal fragment counts across datasets used in this study

Dataset 1 (nucleosomal fragments: 140–180 bp)

Table S1: Summary of nucleosomal fragment counts in Dataset 1

Cell Type	Cell Number	Total Fragments (mean)	Fragments in Open Region (mean)
mESC	203	264,707	13,406
NIH3T3	48	1,906,566	123,151
CD4	288	228,980	14,398

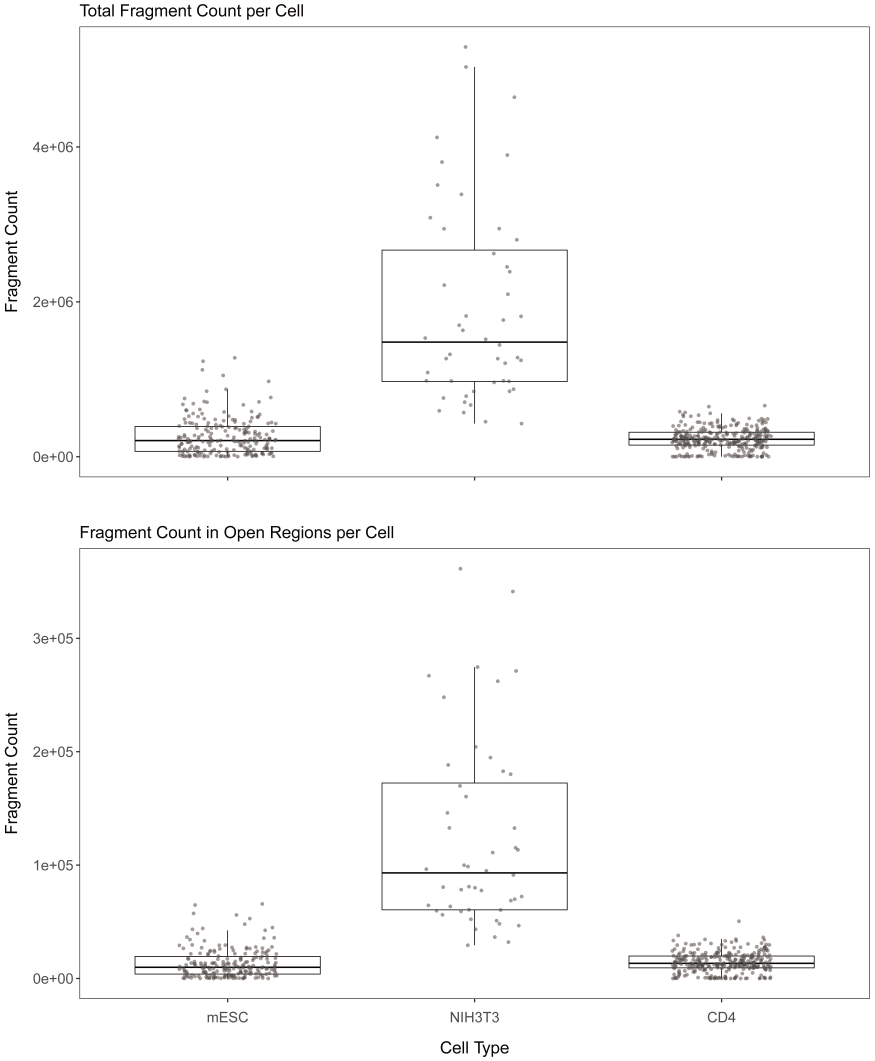

Figure S1: Boxplots showing total fragment counts (top) and fragment counts in open regions (bottom) per cell for mESC, NIH3T3 and CD4 in Dataset 1. Each dot represents a single cell. Open regions are defined as the union of DHS regions (200 bp in width) for each cell type.

Dataset 2 (nucleosomal fragments: 140–180 bp)

Table S2: Summary of nucleosomal fragment counts in Dataset 2

Cell Type	Cell Number	Total Fragments (mean)	Fragments in Open Region (mean)
EILP_KO	250	75,372	327
EILP_WT	331	70,522	307
ILCP_WT	251	59,832	269

Table S3: Summary of nucleosomal fragment counts from pooled single-cell samples in Dataset 2

Cell Type (pooled)	Total Fragments	Fragments in Open Region
Pooled EILP_KO	18,843,000	81,750
Pooled EILP_WT	23,342,782	101,617
Pooled ILCP_WT	15,017,832	67,519

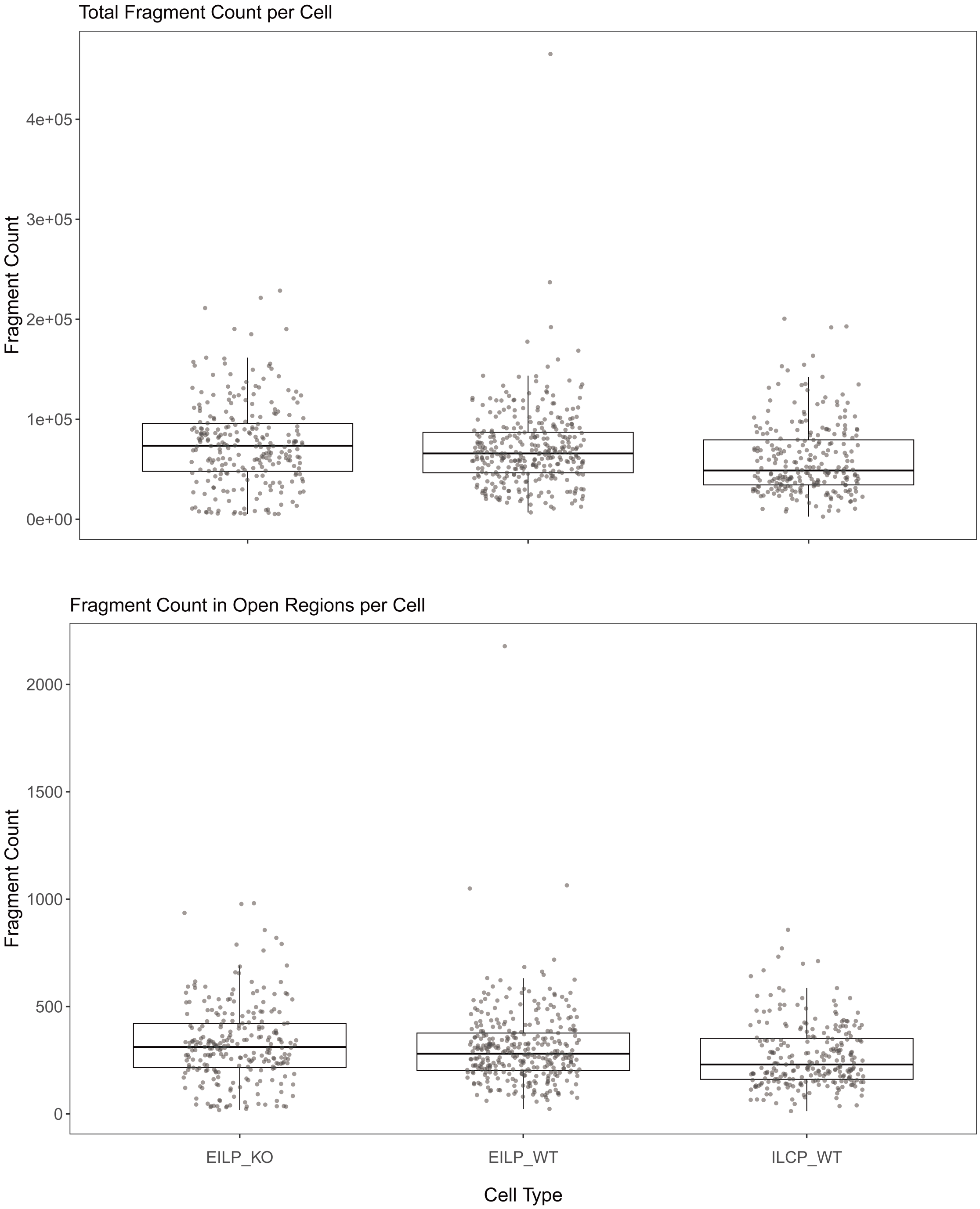

Figure S2: Boxplots showing total fragment counts (top) and fragment counts in open regions (bottom) per cell for EILP_KO, EILP_WT and ILCP_WT in Dataset 2. Each dot represents a single cell. Open regions are defined as the union of DHS regions (200 bp in width) for each cell type.

Dataset 3 (nucleosomal fragments: 140–180 bp)

Table S4: Summary of nucleosomal fragment counts in Dataset 3

Cell Type	Cell Number	Total Fragments (mean)	Fragments in Open Region (mean)
1-cell	5	76,551	181
2-cell	20	579,565	3,047
4-cell	13	1,695,565	5,720
8-cell	22	4,413,300	16,265
morula	31	2,579,024	7,088

Table S5: Summary of nucleosomal fragment counts from pooled single-cell samples in Dataset 3

Cell Type (pooled)	Total Fragments	Fragments in Open Region
Pooled 1-cell	382,755	905
Pooled 2-cell	11,591,300	60,940
Pooled 4-cell	22,042,345	74,360
Pooled 8-cell	97,092,600	357,830
Pooled morula	79,949,744	219,728

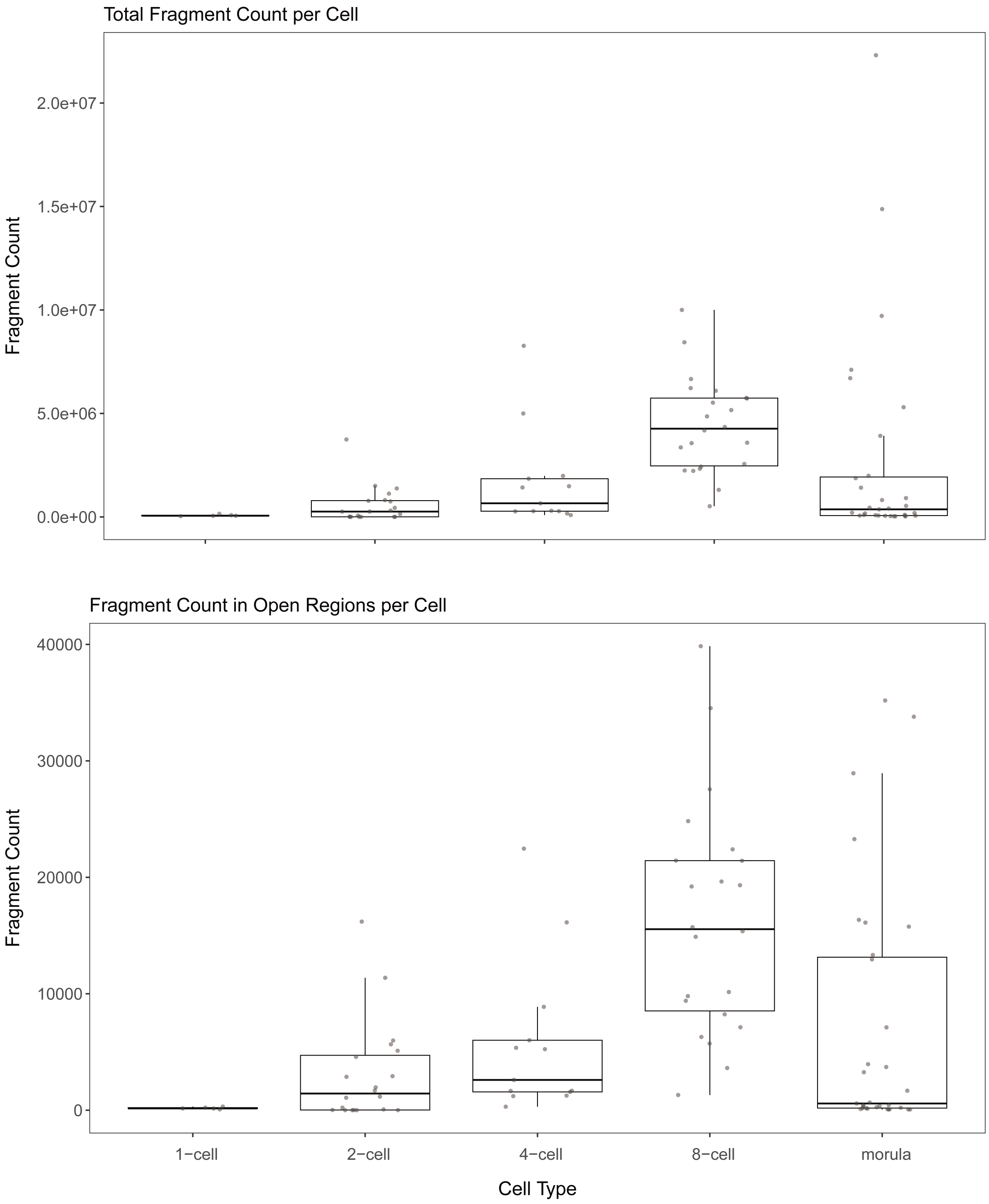

Figure S3: Boxplots showing total fragment counts (top) and fragment counts in open regions (bottom) per cell for 1-cell, 2-cell, 4-cell, 8-cell and morula stages in Dataset 3. Each dot represents a single cell. Open regions are defined as the union of DHS regions (200 bp in width) for each cell type.

Dataset 4 (nucleosomal fragments: 147–294 bp)

Table S6: Summary of nucleosomal fragment counts in Dataset 4

Cell Type	Cell Number	Total Fragments (mean)	Fragments in Region (mean)
CD4	384	6,366	3,409
mCPC	384	29,157	9,374
mESC	384	11,358	3,409
mSF	384	15,837	5,736

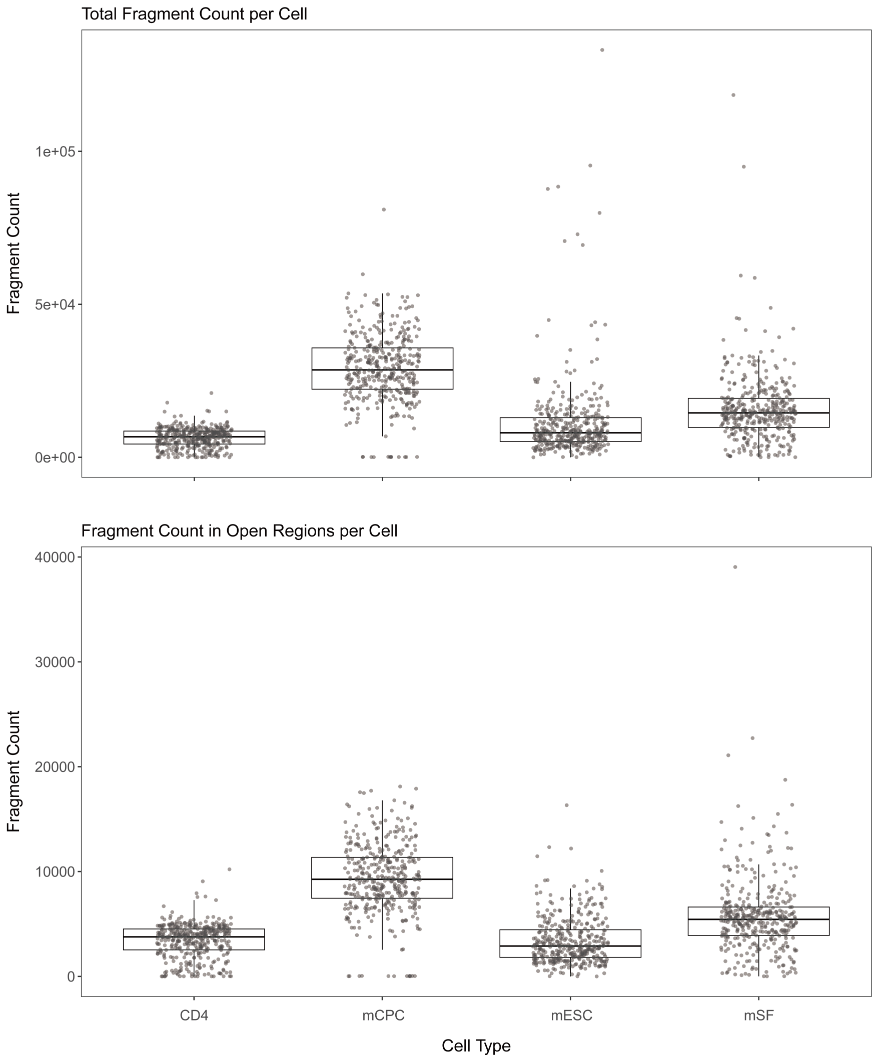

Figure S4: Boxplots showing total fragment counts (top) and fragment counts in open regions (bottom) per cell for CD4, mCPC, mESC and mSF in Dataset 4. Each dot represents a single cell. Open regions were generated by first calling peaks on pooled fragments within each cell type, followed by taking the union across types.

2 Summary of fragment length distribution, total fragment count and FRiP in Dataset 4 (all fragments)

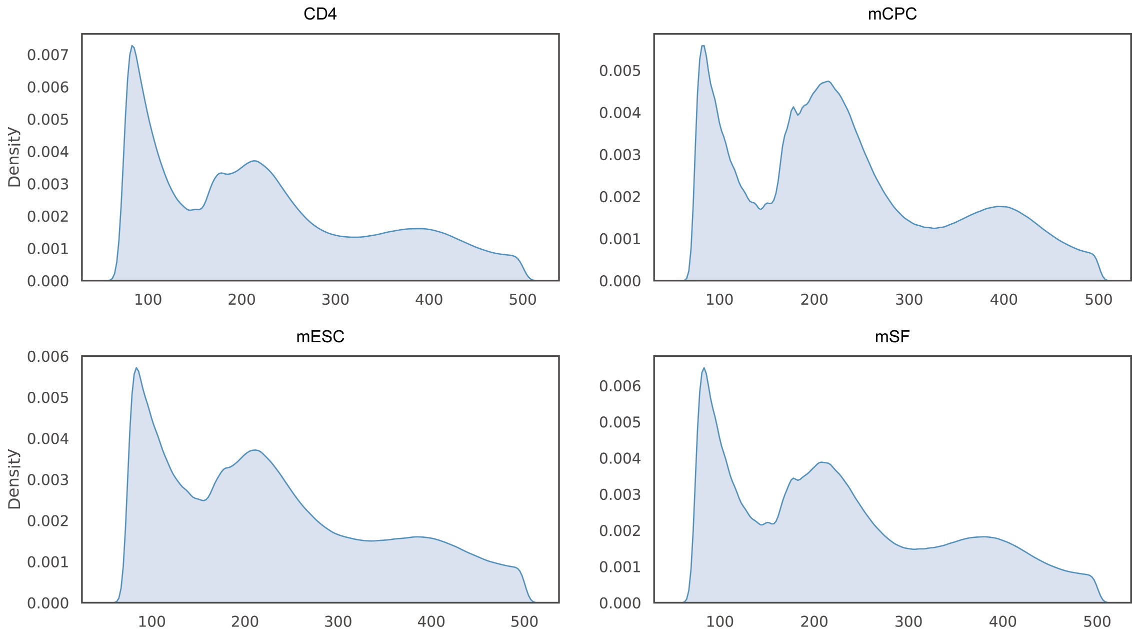

Figure S5: Fragment length density of pooled single-cell fragments grouped by cell type from Dataset 4.

Table S7: Summary of total fragment count and FRiP in Dataset 4

Cell type	Cell Number	Total Fragments (mean)	FRiP (Mean)
CD4	384	15,507	0.6193
mCPC	384	59,562	0.3920
mESC	384	26,692	0.3837
mSF	384	37,531	0.4318

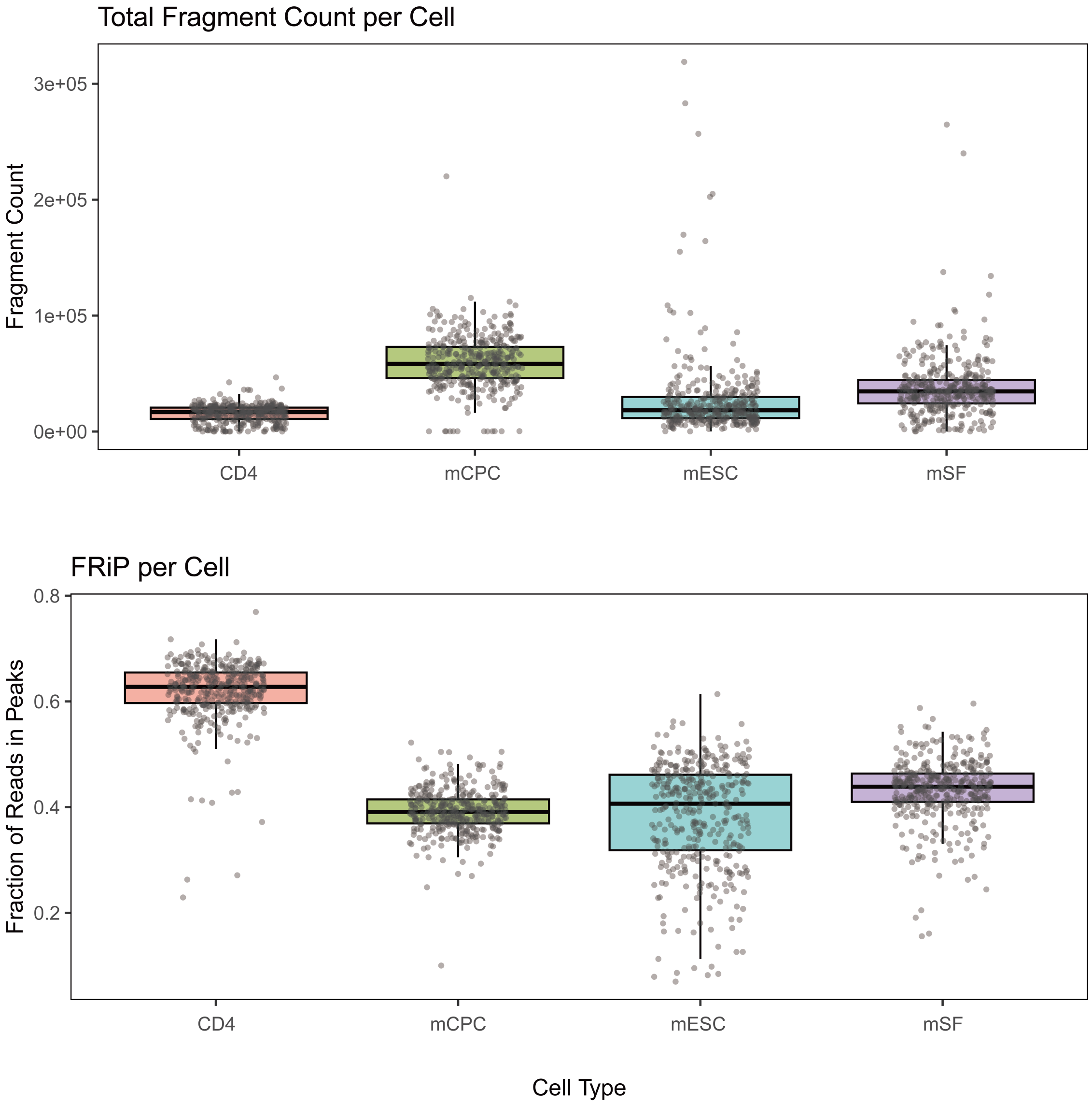

Figure S6: Boxplots showing the total fragment count (top) and fraction of reads in peaks (FRiP, bottom) for each cell across four cell types in Dataset 4. All ATAC-seq fragments, regardless of length, were included. Each dot represents a single cell. The peak set used for FRiP calculation was generated by first calling peaks on pooled fragments within each cell type, followed by taking the union across types.

3 Overall confusion matrices across 16 sampling proportions in robustness testing

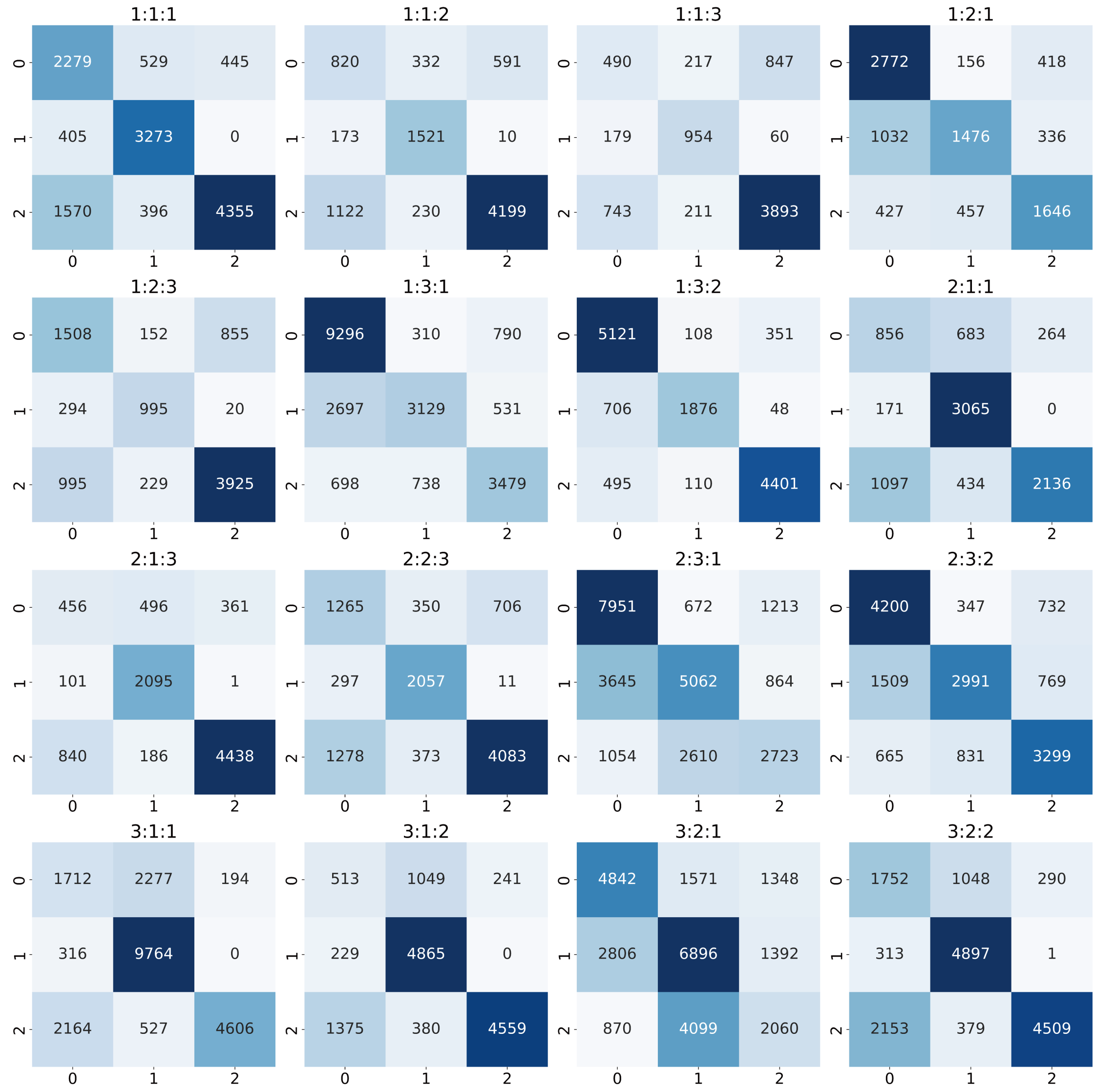

Figure S7: Overall confusion matrices across 16 sampling proportions in robustness testing of scNucMap. Each overall confusion matrix was generated by aggregating the individual confusion matrices from each iteration under a specific sampling proportion. In a confusion matrix, the rows represent the true labels (actual classes) of the samples, while the columns denote the predicted labels (predicted classes) assigned by the model. Each element in the matrix quantifies the number of instances corresponding to a specific combination of true and predicted classes.

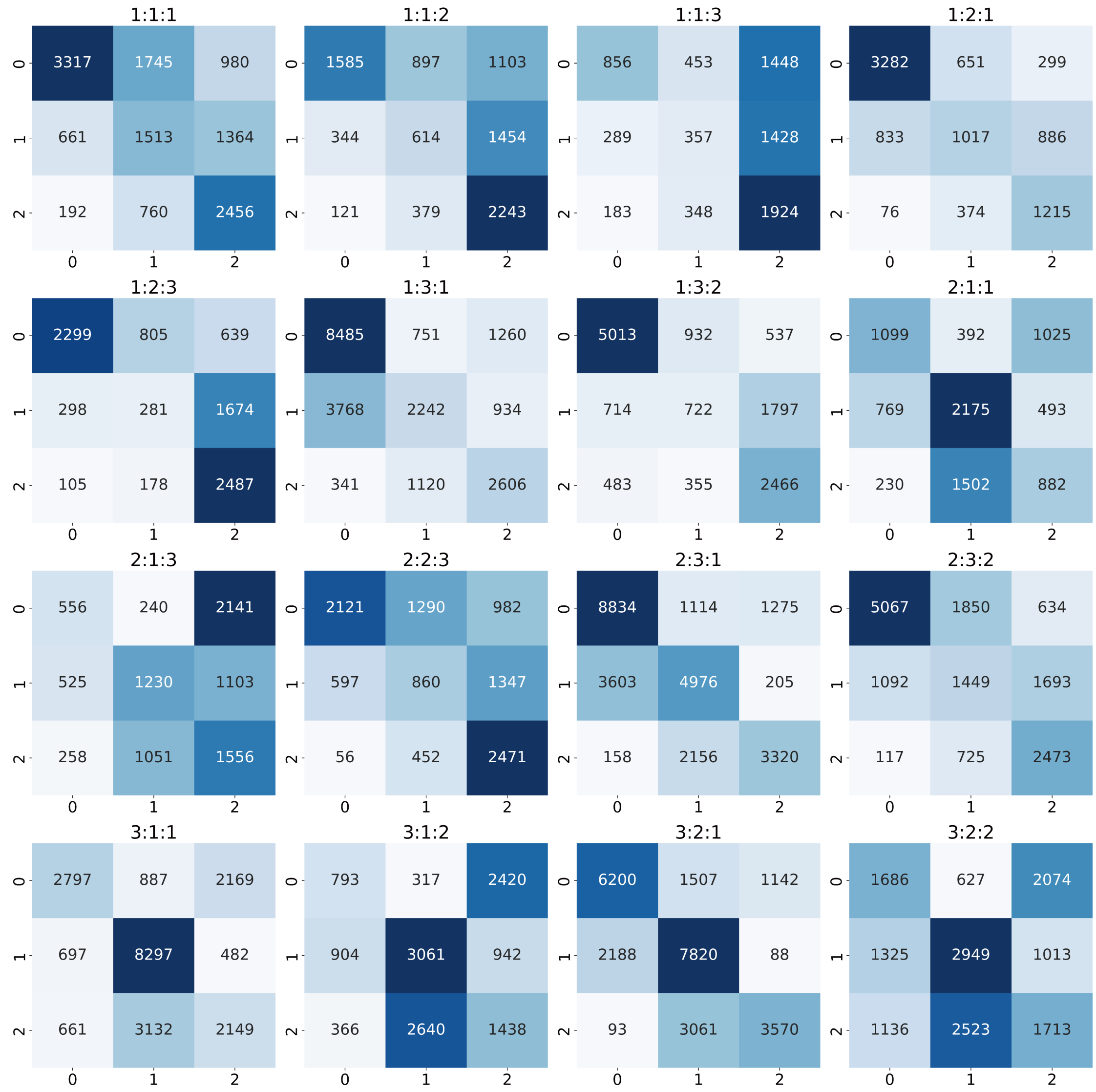

Figure S8: Overall confusion matrices across 16 sampling proportions in robustness testing of chromVAR.

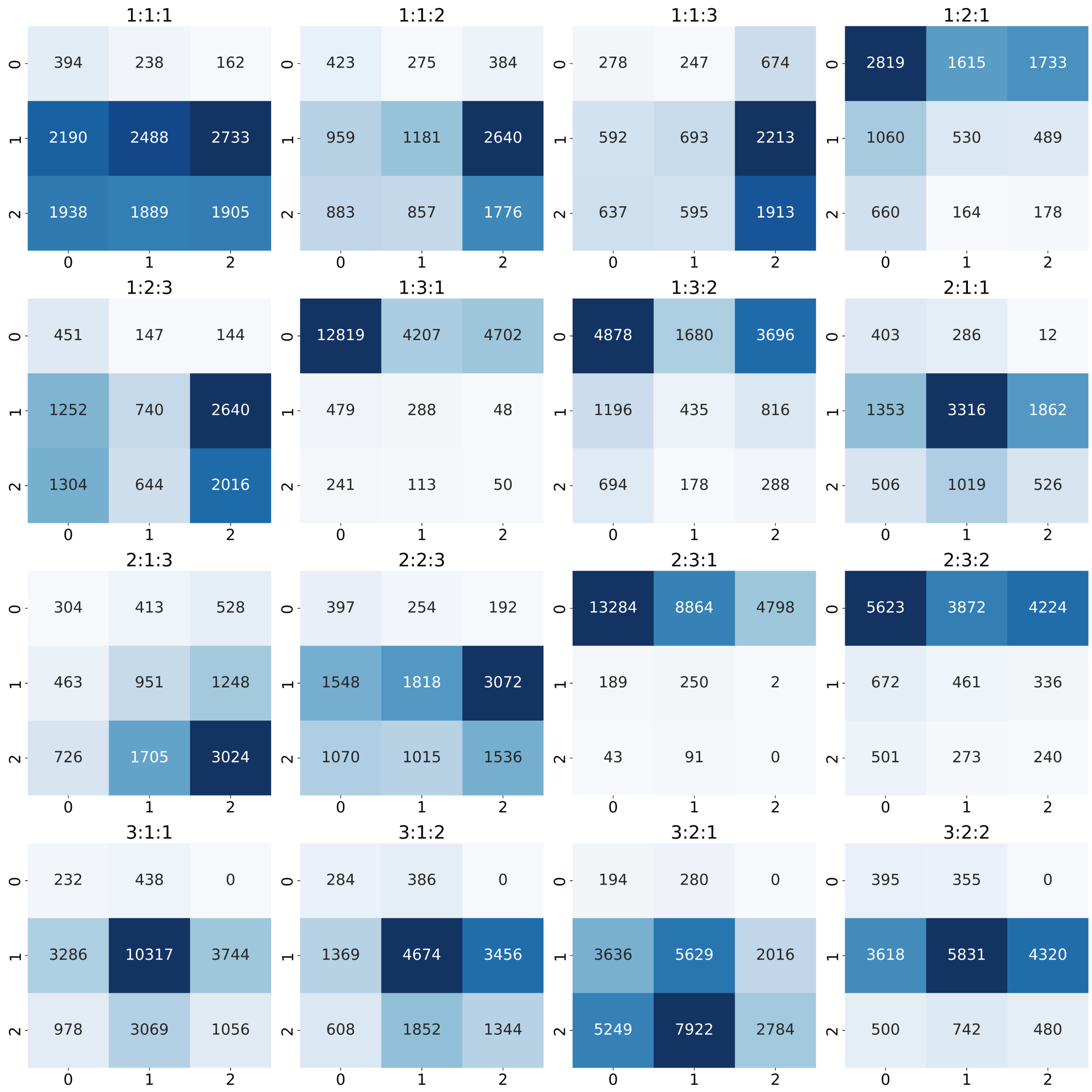

Figure S9: Overall confusion matrices across 16 sampling proportions in robustness testing of Signac.

4 *scNucMap* performance under fragment downsampling

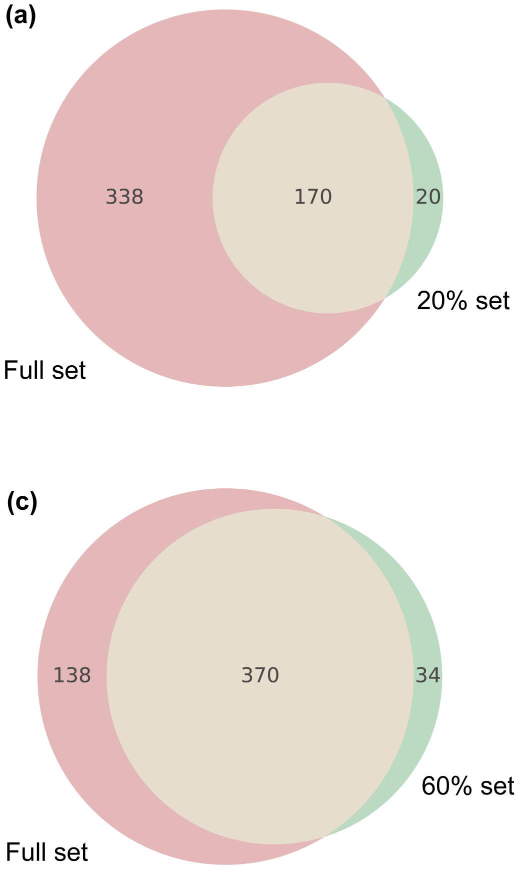

Figure S10: Venn diagrams showing the overlap of motifs identified by the full base dataset and the downsampled datasets. (a) Full base dataset vs. 20% downsampled set. (b) Full base dataset vs. 40% downsampled set. (c) Full base dataset vs. 60% downsampled set. (d) Full base dataset vs. 80% downsampled set.

Table S8: Average Counts of Nucleosomal Fragments (140–180 bp) in NIH3T3, CD4, and mESC Cells from Dataset 1 Across Different Downsampling Levels

**Downsampling Level**	**NIH3T3 (Avg. Reads)**	**CD4 (Avg. Reads)**	**mESC (Avg. Reads)**
20%	381,312	45,795	52,958
40%	762,626	91,591	105,918
60%	1,143,939	137,387	158,877
80%	1,525,252	183,183	211,836
100%	1,906,566	228,979	264,796

Table S9: Performance Metrics Across Downsampling Conditions

**Downsampling Level**	**Overall Accuracy**	**Kappa Coefficient**	**Precision (%)**	**Recall (%)**
20%	0.6634	0.4775	89.47	33.46
40%	0.8884	0.8034	88.17	61.61
60%	0.8948	0.8110	91.58	72.83
80%	0.9367	0.8898	90.00	86.81
100%	0.9499	0.9128	100.00	100.00

Note: We defined the full dataset (100%) as the reference or "true set" of TFs identified by `scNucMap`. For each downsampling level, TFs that overlapped with those in the full dataset were considered true positives (TPs). Precision was calculated as the ratio of TPs to all TFs identified at a given downsampling level, while recall was defined as the ratio of TPs to the total number of TFs identified in the full dataset.

5 Profiles of representative TFs for each cluster in Dataset 1

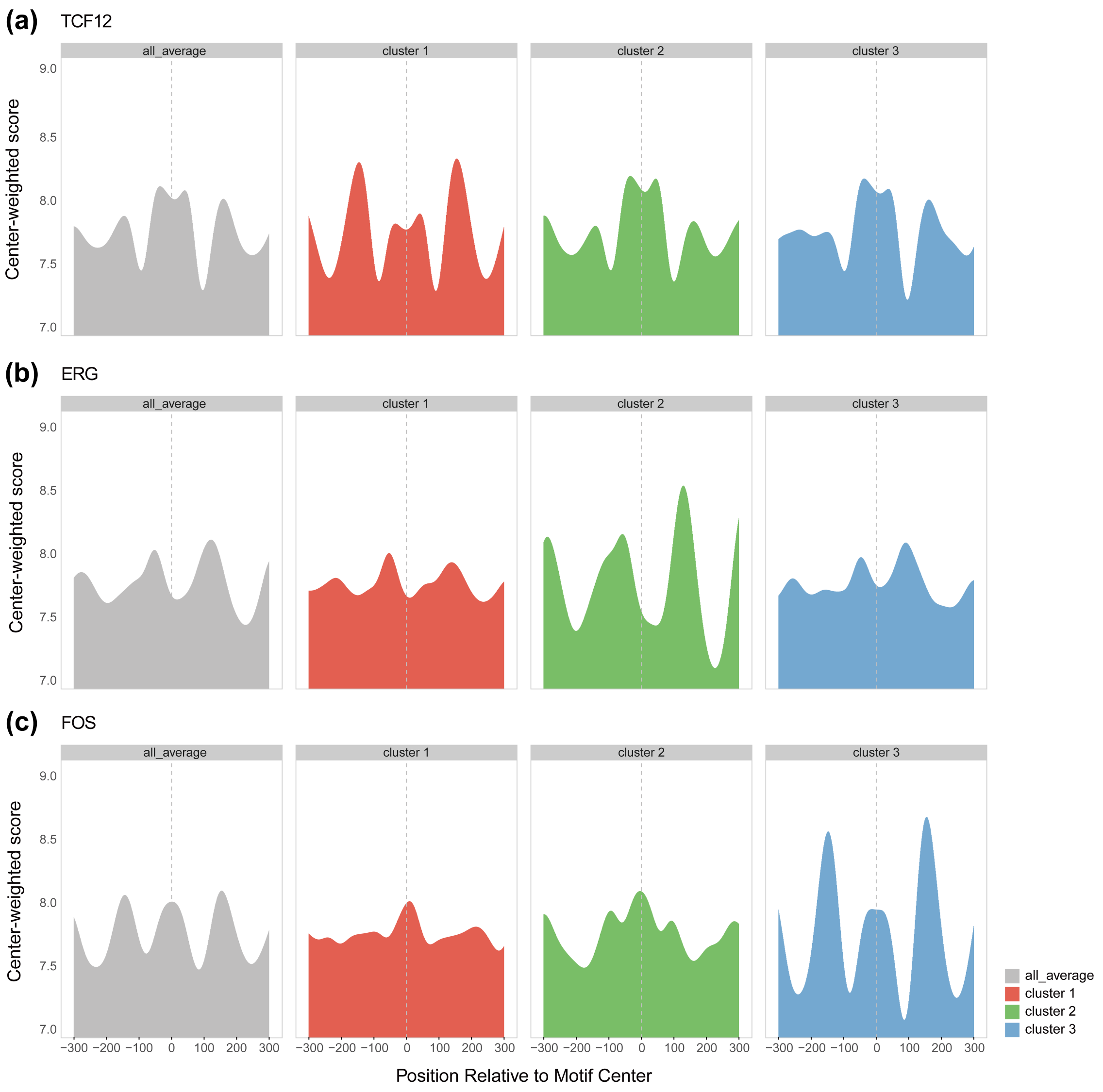

Figure S11: Grey traces represent the average profiles pooled from all samples ("all_average"), while red, green, and blue denote cluster-specific profiles for cluster 1, cluster 2, and cluster 3, respectively.

6 TOBIAS footprinting analysis confirms scNucMap-inferred TF activity patterns

We employed TOBIAS, a footprinting framework that enables genome-wide investigation of TF binding dynamics, to further assess TF binding potential using a mouse embryo ATAC-seq dataset (GEO accession: GSE66581). Since Dataset 3 in our study comprises cells from the 1-cell, 2-cell, 4-cell, 8-cell, and morula stages, while the external ATAC-seq dataset includes only the 2-cell, 4-cell, and 8-cell stages, validation of scNucMap predictions was limited to these overlapping time points. Notably, TF activity of NFYA could not be evaluated due to the absence of 1-cell ATAC-seq data. However, NFYA exhibited consistently higher binding scores than most TFs from the 2-cell to 8-cell stages (Fig. S12a), suggesting a gain in activity at the 2-cell stage that was maintained through to the 8-cell stage.

For the three novel TFs (Pou5f1::Sox2, HES5, and RUNX2) selected for display in Figure 4 of the manuscript, which exhibited gained activity from the 4-cell to 8-cell stage, two of them (Pou5f1::Sox2 and RUNX2) showed increased activity validated independently by TOBIAS analysis (Fig. S12b). These findings provide further support for the validity of scNucMap in capturing stage-specific TF dynamics during early embryogenesis.

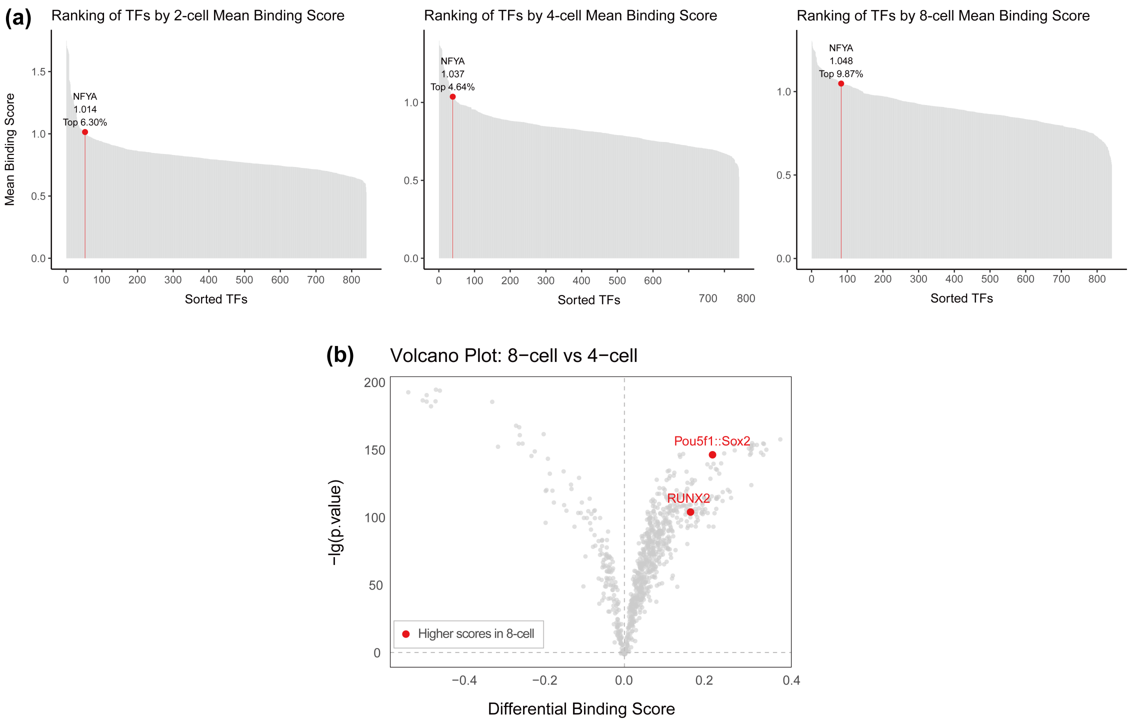

Figure S12: Validation of stage-specific TF activity dynamics using TOBIAS footprinting analysis. (a) TFs were ranked by their mean binding scores at the 2-cell, 4-cell, and 8-cell stages. NFYA (red) exhibited consistently high scores, suggesting a gain in activity at the 2-cell stage that was maintained through to the 8-cell stage. (b) Volcano plot showing TOBIAS-derived differential binding scores (8-cell - 4-cell) and their statistical significance. Pou5f1::Sox2 and RUNX2 exhibited significantly higher binding scores, indicating an increase in activity from the 4-cell to 8-cell stage.

7 All potentially active TFs identified by *scNucMap* in each cluster of Dataset 4

TFs specifically identified by *scNucMap* are marked with ∗ in the "Motif ID" column.

Table S10: List of TFs identified by *scNucMap* in the mESC cluster.

No.	Motif ID	TF Name	p.adjust
1	MA0075.3	PRRX2	0.012
2	MA0079.5	SP1	0.028
3	MA0516.3	SP2	0.0034
4	MA0618.1	LBX1	0.012
5	MA0630.1	SHOX	0.0034
6	MA0654.1	ISX	0.012
7	MA0658.1	LHX6	0.0018
8	MA0661.1	MEOX1	0.0042
9	MA0685.2	SP4	0.028
10	MA0693.3	Vdr	0.012
11	MA0701.2	LHX9	0.012
12	MA0710.1	NOTO	0.00071
13	MA0712.2	OTX2	0.031
14	MA0716.1	PRRX1	0.012
15	MA0720.1	Shox2	0.012
16	MA0721.1	UNCX	0.012
17	MA0740.2	KLF14	0.028
18	MA0741.1	KLF16	0.00027
19	MA0742.2	KLF12	0.022
20	MA0747.1	SP8	0.0042
21	MA0886.1	EMX2	0.028
22	MA0890.1	GBX2	0.028
23	MA0892.1	GSX1	9.3e-05
24	MA0903.1	HOXB3	0.00071
25	MA1463.1	ARGFX	0.028
26	MA1522.1	MAZ	0.028
27	MA1540.2	NR5A1	0.028
28	MA1564.1	SP9	0.0042
29	MA1587.1	ZNF135	0.028
30	MA1603.1	Dmrt1	0.012
31	MA1627.1	Wt1	0.028
32	MA1630.2	ZNF281	0.0042
33	MA1653.1	ZNF148	0.0017
34	MA1710.1	ZNF257	0.028
35	MA1715.1	ZNF707	0.028
36	MA1724.1	Rfx6	0.00065
37	MA1961.1	PATZ1	0.0034
38	MA1973.1	ZKSCAN3	0.028
39	MA1981.1	ZNF530	0.012
40	MA1987.1	ZNF701	1.2e-06
41	∗MA0030.1	FOXF2	0.0042
42	∗MA0048.2	NHLH1	0.028
43	∗MA0073.1	RREB1	9.2e-09

Continued on next page

No.	Motif ID	TF Name	p.adjust
44	∗MA0091.1	TAL1::TCF3	9.3e-05
45	∗MA0112.3	ESR1	0.028
46	∗MA0119.1	NFIC::TLX1	0.044
47	∗MA0139.1	CTCF	0.0003
48	∗MA0149.1	EWSR1-FLI1	7.1e-08
49	∗MA0162.4	EGR1	0.0042
50	∗MA0164.1	Nr2e3	0.011
51	∗MA0481.3	FOXP1	0.0042
52	∗MA0494.1	Nr1h3::Rxra	0.0034
53	∗MA0506.2	Nrf1	0.028
54	∗MA0675.1	NKX6-2	0.012
55	∗MA0732.1	EGR3	0.0012
56	∗MA0752.1	ZNF410	0.028
57	∗MA0753.2	ZNF740	1.1e-07
58	∗MA0860.1	Rarg	0.0017
59	∗MA0863.1	MTF1	0.0034
60	∗MA0910.2	HOXD8	0.044
61	∗MA1102.2	CTCFL	0.0012
62	∗MA1107.2	KLF9	9.3e-05
63	∗MA1108.2	MXI1	0.00064
64	∗MA1109.1	NEUROD1	0.028
65	∗MA1118.1	SIX1	0.019
66	∗MA1119.1	SIX2	0.0028
67	∗MA1125.1	ZNF384	9.2e-09
68	∗MA1155.1	ZSCAN4	5.2e-17
69	∗MA1467.2	Atoh1	0.031
70	∗MA1502.1	HOXB8	0.044
71	∗MA1528.1	NFIX	0.0034
72	∗MA1549.1	POU6F1	0.041
73	∗MA1570.1	TFAP4	0.00076
74	∗MA1578.1	VEZF1	0.0034
75	∗MA1583.1	ZFP57	0.011
76	∗MA1588.1	ZNF136	0.031
77	∗MA1593.1	ZNF317	0.0012
78	∗MA1602.1	ZSCAN29	0.0017
79	∗MA1631.1	ASCL1	0.028
80	∗MA1642.1	NEUROG2	0.00011
81	∗MA1713.1	ZNF610	0.028
82	∗MA1721.1	ZNF93	9.2e-09
83	∗MA1723.1	PRDM9	2.9e-12
84	∗MA1730.1	ZNF708	0.028
85	∗MA1731.1	ZNF768	0.00039
86	∗MA1929.1	CTCF	1.2e-16
87	∗MA1930.1	CTCF	0.012
88	∗MA1966.1	TFAP4::ETV1	0.0042
89	∗MA1969.1	THRA	9.2e-08

Table S11: List of TFs identified by *scNucMap* in the CD4 cluster.

No.	Motif ID	TF Name	p.adjust
1	MA0076.2	ELK4	2.6e-05
2	MA0603.1	Arntl	0.00014
3	MA0631.1	Six3	0.044
4	MA0645.1	ETV6	0.0023
5	MA0750.2	ZBTB7A	0.037
6	MA0761.2	ETV1	0.0013
7	MA0772.1	IRF7	0.044
8	MA1464.1	ARNT2	0.0023
9	MA1484.1	ETS2	0.033
10	MA1708.1	ETV7	0.044
11	MA1933.1	ELK1::SREBF2	0.024
12	∗MA0018.4	CREB1	0.0023
13	∗MA0060.3	NFYA	0.00071
14	∗MA0461.2	Atoh1	0.044
15	∗MA0502.2	NFYB	0.039
16	∗MA0604.1	Atf1	0.00012
17	∗MA0605.2	ATF3	0.00028
18	∗MA0609.2	CREM	0.00019
19	∗MA0638.1	CREB3	0.025
20	∗MA0656.1	JDP2	0.00071
21	∗MA0834.1	ATF7	0.045
22	∗MA1126.1	FOS::JUN	0.0013
23	∗MA1127.1	FOSB::JUN	0.004
24	∗MA1129.1	FOSL1::JUN	0.044
25	∗MA1131.1	FOSL2::JUN	2.4e-05
26	∗MA1133.1	JUN::JUNB	0.00071
27	∗MA1139.1	FOSL2::JUNB	0.022
28	∗MA1140.2	JUNB	0.002
29	∗MA1143.1	FOSL1::JUND	0.00012
30	∗MA1145.1	FOSL2::JUND	0.004
31	∗MA1475.1	CREB3L4	5.1e-06
32	∗MA1517.1	KLF6	0.019
33	∗MA1644.1	NFYC	0.00027
34	∗MA1951.1	FOS	0.013

Table S12: List of TFs identified by *scNucMap* in the mCPC cluster.

No.	Motif ID	TF Name	p.adjust
1	MA0139.1	CTCF	1.3e-55
2	MA1929.1	CTCF	2e-09
3	MA1930.1	CTCF	1.5e-26
4	∗MA0028.2	ELK1	0.0001
5	∗MA0098.3	ETS1	0.012
6	∗MA0156.3	FEV	0.0064
7	∗MA0475.2	FLI1	0.00086
8	∗MA0641.1	ELF4	0.0016
9	∗MA0759.2	ELK3	0.003
10	∗MA0760.1	ERF	1.1e-05
11	∗MA0763.1	ETV3	0.0032
12	∗MA0764.3	ETV4	1.5e-06
13	∗MA0765.3	ETV5	0.0016
14	∗MA1139.1	FOSL2::JUNB	0.048
15	∗MA1145.1	FOSL2::JUND	0.0055
16	∗MA1483.2	ELF2	0.02
17	∗MA1931.1	ELK1::HOXA1	0.0058
18	∗MA1957.1	HOXB2::ELK1	0.0054

.

Table S13: List of TFs identified by *scNucMap* in the mSF cluster.

No.	Motif ID	TF Name	p.adjust
1	MA0089.2	MAFG::NFE2L1	0.013
2	MA0099.3	FOS::JUN	6.6e-25
3	MA0462.2	BATF::JUN	1.8e-20
4	MA0476.1	FOS	4.2e-25
5	MA0477.2	FOSL1	3.4e-21
6	MA0478.1	FOSL2	6.1e-29
7	MA0489.2	Jun	1.1e-28
8	MA0490.2	JUNB	7.4e-16
9	MA0491.2	JUND	1.2e-16
10	MA0496.3	MAFK	0.016
11	MA0655.1	JDP2	9.8e-15
12	MA0835.2	BATF3	1.8e-20
13	MA0841.1	NFE2	6.2e-10
14	MA1101.2	BACH2	7.4e-16
15	MA1128.1	FOSL1::JUN	6.7e-23
16	MA1130.1	FOSL2::JUN	1.5e-22
17	MA1132.1	JUN::JUNB	9.9e-15
18	MA1134.1	FOS::JUNB	3.5e-22
19	MA1135.1	FOSB::JUNB	2.5e-22
20	MA1137.1	FOSL1::JUNB	1.8e-16
21	MA1138.1	FOSL2::JUNB	4e-18
22	MA1141.1	FOS::JUND	2.5e-22
23	MA1142.1	FOSL1::JUND	3.6e-13
24	MA1144.1	FOSL2::JUND	1.2e-20
25	MA1633.2	BACH1	3.9e-20
26	MA1634.1	BATF	3.4e-21
27	MA1928.1	BNC2	3.5e-31
28	MA1988.1	Atf3	2e-25

8 Performance evaluation of the initial version of *scNucMap*

scNucMap provides two computing modes, each optimized for different hardware capabilities, allowing users to choose the mode that best suits their system.

- **Standard**: Prioritizes speed when sufficient memory is available.
- **Lite**: Prioritizes memory efficiency for large datasets or limited computing environments.

This dual-mode design allows users to flexibly adapt the method to various computational constraints. To construct a base dataset with approximately 0.1 million nucleosomal fragments per cell, we downsampled the mESC cell samples (n = 203) from Dataset 1 to 40% of the original fragments, resulting in an average of 105,918 nucleosomal fragments per cell, with the highest number of fragments in a single cell being 2,116,358. By replicating this base dataset, we generated additional testing datasets at specific fold-change levels.

All tests were performed on a single HPC node equipped with dual Intel Xeon Gold 6342 CPUs (2.80 GHz base frequency, 48 cores, 96 threads) and 503 GB of RAM.

Table S14: Runtime and memory usage of *scNucMap* across dataset sizes and modes (48 threads)

**Number of Cells**	**Runtime (hh:mm:ss)**		**Peak RAM (GB)**	
	**Standard**	**Lite**	**Standard**	**Lite**
203	00:16:35	00:19:00	113.04	1.70
2,030	00:44:04	02:09:32	113.04	1.72
4,060	01:05:09	04:28:17	113.10	1.73
10,150	02:45:19	11:01:25	113.13	1.77
20,300	05:17:32	21:42:44	113.23	1.84

Table S15: Runtime and memory usage of *scNucMap* for a million-cell dataset (96 threads)

**Number of Cells**	**Avg. reads/cell**	**Max reads (single)**	**Runtime (hh:mm:ss)**	**Peak RAM**
1,015,000	105,918	2,116,358	75:42:18	122.60 GB
